# Addressing skepticism of the critical brain hypothesis

**DOI:** 10.3389/fncom.2022.703865

**Published:** 2022-09-15

**Authors:** John M. Beggs

**Affiliations:** ^1^Department of Physics, Indiana University Bloomington, Bloomington, IN, United States; ^2^Program in Neuroscience, Indiana University Bloomington, Bloomington, IN, United States

**Keywords:** neuronal avalanche, criticality, hypothesis, phase transition, temporal correlations

## Abstract

The hypothesis that living neural networks operate near a critical phase transition point has received substantial discussion. This “criticality hypothesis” is potentially important because experiments and theory show that optimal information processing and health are associated with operating near the critical point. Despite the promise of this idea, there have been several objections to it. While earlier objections have been addressed already, the more recent critiques of Touboul and Destexhe have not yet been fully met. The purpose of this paper is to describe their objections and offer responses. Their first objection is that the well-known Brunel model for cortical networks does not display a peak in mutual information near its phase transition, in apparent contradiction to the criticality hypothesis. In response I show that it does have such a peak near the phase transition point, provided it is not strongly driven by random inputs. Their second objection is that even simple models like a coin flip can satisfy multiple criteria of criticality. This suggests that the emergent criticality claimed to exist in cortical networks is just the consequence of a random walk put through a threshold. In response I show that while such processes can produce many signatures criticality, these signatures (1) do not emerge from collective interactions, (2) do not support information processing, and (3) do not have long-range temporal correlations. Because experiments show these three features are consistently present in living neural networks, such random walk models are inadequate. Nevertheless, I conclude that these objections have been valuable for refining research questions and should always be welcomed as a part of the scientific process.

## Introduction

*“I am that gadfly which God has attached to the state, all day long*…*arousing and persuading and reproaching*…*You will not easily find another like me.”*

-Socrates, in Plato’s Apology

The “criticality hypothesis” states that the brain operates near a phase transition point for optimal information processing (Beggs, 2008; Chialvo, 2010; Shew and Plenz, 2013; Cocchi et al., 2017). The origins of this idea trace back over several decades and derive from many investigators: Kauffman (1969); Wilson and Cowan (1972); Kelso (1984); Freeman (1987); Dunkelmann and Radons (1994); Bienenstock (1995); Herz and Hopfield (1995); Bak (1996); Chialvo and Bak (1999); De Carvalho and Prado (2000); Greenfield and Lecar (2001); Linkenkaer-Hansen et al. (2001); Eurich et al. (2002); Worrell et al. (2002).

To illustrate this hypothesis, consider the three possible ways that activity could propagate in a neural network. First, it could be damped so that activity in one neuron would, on average, lead to activity in less than one neuron in the next time step. This is the subcritical phase. To quantify this, we can use the branching ratio, σ, which gives the average number of descendant neurons from a single active parent neuron. Thus, the subcritical phase has a branching ratio of less than one (σ < 1). Second, activity could be amplified so that one active neuron would, on average, activate more than one neuron in the next time step. This is the supercritical phase, characterized by a branching ratio greater than one (σ > 1). Third, activity could be balanced so that one active neuron would, on average, activate one neuron in the next time step. This is a critical point, poised between the damped and amplified phases, and characterized by a branching ratio exactly equal to one (σ = 1). When a network operates near a critical point (σ≈ 1), it produces avalanches of neural activity whose size distributions approximately follow power laws (Beggs and Plenz, 2003; Petermann et al., 2009; Shew et al., 2015; Ponce-Alvarez et al., 2018).

In addition, near a critical point, information processing functions like the dynamic range (Kinouchi and Copelli, 2006; Shew et al., 2009) and the amount of information that can be transmitted through a network (Greenfield and Lecar, 2001; Beggs and Plenz, 2003; Shew et al., 2011) are maximized. Very briefly, this is because communication between neurons is extremely weak in the subcritical phase when activity dies out, and it is saturated in the supercritical phase when it is amplified (Beggs, 2008; Shew and Plenz, 2013). Between these extremes, near a critical point, information transmission is greatest. Both models and experimental data are consistent with this picture. Other functions that are predicted to be optimized near the critical point include computational power (Bertschinger and Natschlager, 2004; Legenstein and Maass, 2007), information storage (Haldeman and Beggs, 2005), sensitivity to changes in inputs (Williams-Garcia et al., 2014), and controllability of dynamics without instability (Chialvo et al., 2020; Finlinson et al., 2020). Many of these functions are nicely reviewed in Shew and Plenz (2013).

Evidence for nearly critical dynamics now has been found in a wide range of species including zebrafish (Ponce-Alvarez et al., 2018), turtles (Shew et al., 2015), rodents (Fontenele et al., 2019), monkeys (Petermann et al., 2009), and humans (Priesemann et al., 2013; Shriki et al., 2013).

In this paper we will cover ideas and models that are positioned as rivals to the criticality hypothesis. Such rivals are extremely useful, as they become dialog partners, helping us to refine what we really mean when we say a network is critical, or what falsifiable predictions need to be addressed in experiments. These rivals may even be right, and objective science should always leave open the possibility that a hypothesis, however beautiful or psychologically dear, might be wrong. In the interest of such rational discussion, and to guard against becoming too subjective, it is vitally important to examine these alternative ideas–to not kill the gadfly but let it bite. One way to do this is by presenting the opposition in the strongest way possible, and not as a weakened straw man that can be easily knocked down. What are the best counterarguments? Can the criticality hypothesis meet them, or does it survive only if opposing ideas arrive pre-damaged before doing battle?

Let us overview several waves of criticism so far. Briefly, an early wave argued that many neural data sets that were claimed to follow power laws did not pass appropriate statistical tests. The field responded by consistently applying more statistical rigor. This revealed that while some neural data sets were not best fit by power laws, many in fact were (Klaus et al., 2011; Bellay et al., 2015; Shew et al., 2015; Timme et al., 2016; Ponce-Alvarez et al., 2018). Another early issue raised as criticism was that several non-critical processes, like successive fragmentation or random combinations of exponentials, could also produce power laws (Reed and Hughes, 2002; Mitzenmacher, 2004). Here, the field responded by developing additional tests for criticality that went beyond power laws. These included the exponent relation (Sethna et al., 2001; Friedman et al., 2012), avalanche shape collapse, evidence of long-range temporal correlations (Hardstone et al., 2012) and a more accurate measure of the branching ratio (Wilting and Priesemann, 2018), improvements that are now widely adopted. A summary of many of these critiques and how they were met can be found in Beggs and Timme (2012). Toolboxes for implementing these improvements can be found in Hardstone et al. (2012); Ihlen (2012); Alstott et al. (2014); Marshall et al. (2016); Spitzner et al. (2020).

Another issue that has been raised is that there may be no critical phase transition at all. For example, (Martinello et al., 2017) argue that neutral drift can account for many of the observations seen in experiments, like scale-free power laws. However, this idea of neutral drift is difficult to reconcile with experimental evidence of homeostasis actively working to restore perturbed networks back toward the critical point (Meisel et al., 2013; Shew et al., 2015; Ma et al., 2019; Meisel, 2020).

A more recent wave of criticism has come through the work of Touboul and Destexhe (Touboul and Destexhe, 2017; Destexhe and Touboul, 2021). Their first claim is that operating near the critical point does not necessarily optimize information processing. To demonstrate their point, they investigated the well-known model of spiking cortical networks developed by Brunel (2000). In their hands, they showed that response entropy (which can also be called the information capacity) did not have a peak, but rather a step-like transition, as the model was moved from the synchronous irregular (SI) phase of firing to the asynchronous irregular (AI) phase (Touboul and Destexhe, 2017). The lack of a peak in information capacity, they claim, demonstrates that operating near a phase transition does not optimize information processing. This would be a clear contradiction of the criticality hypothesis. Their second claim is that when a Brunel model with no internal synaptic connections is driven by a very simple random process like a coin flip or a modified random walk, it can show many signatures of criticality (Touboul and Destexhe, 2017; Destexhe and Touboul, 2021). If random noise passed through a threshold can mimic the power laws and exponent relation seen in the data, then why do we need to hypothesize that the apparent criticality in living neural networks is anything more than this? The contradiction with the criticality hypothesis here is somewhat less obvious. The claim is that signatures of criticality present in living neural networks are not a result of collective interactions among neurons. In other words, neuronal criticality is not emergent like the criticality observed in an ensemble of water molecules or in a sample of iron.

Before going further, let us revisit and update the criticality hypothesis to explain it in more detail. This will allow us to respond to the two critiques more specifically. I take the criticality hypothesis to mean the following:

*When a network of neurons operates near a critical phase transition point, multiple information processing functions (e.g., information transmission and storage, dynamic range, susceptibility to inputs and computational power) are simultaneously optimized through collective interactions among neurons.*

I want to emphasize three facets of this hypothesis. First, the network needs to be near a critical point. This will lead us to consider multiple signatures of criticality. Second, near a critical point, information processing will be optimized. This will lead us to search for a peak in information transmission (objection 1). Third, both a critical point and optimal information processing emerge through the collective interactions of many neurons in a network. This will lead us to distinguish collective models with interacting neurons from simple, random walk models without interactions (objection 2).

In what follows, I first review the criteria that we will apply to determine if a system is operating near the critical point. I next explain the claims of Touboul and Destexhe with more detail so they can be assessed by the reader. I then present computational demonstrations to challenge their claims. To streamline the presentation, methodological details of these simulations are contained in the Supplementary material. I conclude by noting that their arguments against the criticality hypothesis do not constitute a refutation. However, they are still very useful in refining our interpretations of criticality experiments.

## Signatures of criticality

### An intuitive description of criticality

In the most simplified terms, a system that exhibits criticality must be a tunable system. For example, at a particular pressure, water can be tuned from its gas phase to its liquid phase as the temperature is reduced. To take another example, when a piece of iron is cooled, it is tuned from a phase where its spins were pointing equally up and down, to a phase where they are all pointing in the same direction. Similarly, as the strength of synaptic connections is increased, a neural network can be tuned from a phase where neurons are firing independently to a phase where they are all firing synchronously. In these examples, the variable that tunes the system is called the control parameter; for water and iron this is the temperature, while for neural networks it is the connection strengths.

Notice also the differences between the two phases. One phase is random, high in energy and has symmetry, while the other is ordered, lower in energy and is associated with some breaking of symmetry. For the water example, the high energy phase is the gas, where molecules are equally likely to be in any location within the volume. The low energy phase is the liquid, where the molecules coalesce into a reduced volume. For the iron example, the high energy phase consists of spins pointing equally up and down, while the low energy phase breaks this symmetry and has all the spins pointing the same way. For neural networks, disconnected, randomly firing neurons visit a broad range of network states, while strongly coupled synchronous neurons are confined to a relatively small region of state space.

The critical point in such tunable systems occurs right between these phases, when the control parameter is at its critical value. At the critical point, these systems are a mixture of randomness and order. They have neither the complete symmetry associated with randomness nor the order associated with symmetry-breaking. Rather, they have both variety and structure across all scales.

The most common way to identify this scale-invariant structure has been to observe power law distributions. At the critical point, spatial and temporal correlations fall off slowly with power law tails; distributions of avalanche sizes and durations also follow a power law. When the system is sufficiently far away from the critical point, power law distributions disappear. The power laws at criticality indicate that spatial and temporal correlations diverge–which means that their average values become infinite. This also allows information to pass through the system most readily at the critical point. As a result, plots of mutual information or temporal correlations should show a peak when the control parameter is tuned to its critical value.

As mentioned earlier, power laws by themselves are insufficient to determine whether a system operates near a critical point–additional criteria are needed. Some phenomena like successive fractionation and the summation of many exponential processes can produce power law distributions. Yet these are not clearly tunable systems that exhibit phase transitions or symmetry breaking. How then can we distinguish between critical and non-critical systems that both produce power laws?

### Moving beyond power laws

Fortunately, critical phase transitions have been studied extensively in physics, and the literature there provides guidance on how to proceed. In a seminal paper published in 2001, Sethna et al. (2001) argued that to move beyond power laws alone, we should examine scaling functions. Perhaps the easiest way to describe a scaling function is by giving an example of it from neural data.

**FIGURE 1:**
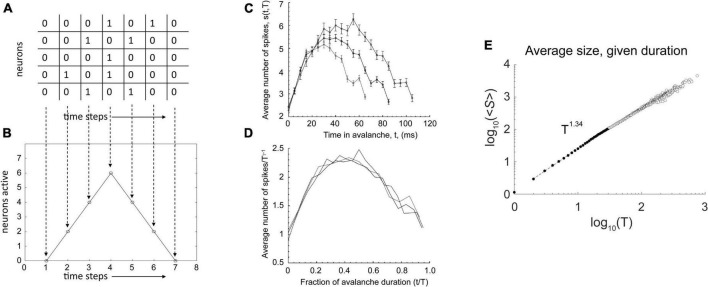
Additional signatures of criticality. **(A)** A toy raster plot where spikes are represented by 1 s and no activity by 0 s. Here, five neurons are recorded over seven time bins. An avalanche is a sequence of consecutively active time bins, bracketed by time bins with no activity. **(B)** The raster can be used to construct the avalanche shape, which is just the number of active neurons at each time bin. Here, we have a tent shape. **(C)** Average avalanche shapes for three different lengths, taken from microelectrode array recordings of cortical slice cultures. Here, the shapes are like inverted parabolas. **(D)** With appropriate rescaling of avalanche duration and height (explained in text), these avalanche shapes collapse on top of each other, demonstrating that the inverted parabola is a scaling function for this network. Such a scaling function is expected to exist only very close to a critical point. **(E)** Avalanche size is related to avalanche duration by a power law. The *y*-axis is the average avalanche size, ≪*cps*:*it* > *S* < /*cps*:*it*≫, for a given duration, T. The *x*-axis is the avalanche duration, T. Because these data nearly follow a straight line in log-log axes, we can say that they approximate a power law. We can estimate the exponent, γ, of the power law by the slope of the line. In this case, it is 1.34. Thus, avalanche size scales with the duration according to this relationship: ≪*cps*:*it* > *S* < /*cps*:*it*≫(T) ∼ T^1.34^. A scaling relationship between size and duration is necessary for avalanche shape collapse. Data from Fosque et al. (2021). The portion of the data used for estimating the power law is shown as filled circles with dashed line. Fitting was performed using software from Marshall et al. (2016). Panels **(C,D)** adapted from Friedman et al. (2012).

Consider a toy raster plot of activity from a neural network, shown in Figure 1A. In the avalanche there, we can plot the number of neurons active in each time bin to produce an avalanche shape that describes how the activity unfolds over time (Figure 1B). When this is done for an actual data set, we see shapes that look like inverted parabolas (Figure 1C). More generally, such shapes could be semicircular or skewed parabolas (Laurson et al., 2013). No matter what the shapes, if they are rescaled, they can be made to collapse onto each other (Figure 1D) in systems near the critical point. This rescaling is done for both the time and the height of the avalanche. For time, each avalanche is divided by its duration, so that all avalanche shapes to be compared will have a length of 1. For height, each avalanche is rescaled by dividing it by its maximum height, so all avalanches will have the same height. To get the maximum height, h_max_, we realize that the size S of the avalanche (its area under the curve) is proportional to (∝) its duration T times h_max_. This means h_max_ is proportional to S divided by T: h_max_ ∝ S/T. As we see in Figure 1E, there is a scaling relation (∼) between avalanche size and duration: S ∼ T^γ^. Thus, we have h_max_ ∝ T^γ–1^/T = T^γ–1^. If we divide each average avalanche shape by T^γ–1^, then they will all have the same heights. Note that this is possible only if they follow a scaling relation like the one shown in Figure 1E.

When this avalanche shape collapse occurs, it shows that the avalanche shapes are all similar, no matter what their sizes. In other words, they are fractal copies of each other, each merely being a version of an inverted parabola that is either scaled up for larger avalanches or scaled down for smaller ones. Because all the average avalanches can be made to follow this shape by rescaling, it is called a universal scaling function.

You might think that such a function should always occur, but it does not. For example, consider what would happen if a network produced tent-like avalanche shapes (Figure 1B), but with different slopes. Say the longer avalanches had shallower slopes and the shorter avalanches has steeper ones. While it might be possible to rescale all of them to the same length, they would not then all have the same heights, and so they would not collapse on top of each other. Likewise, one could rescale all their heights, but then they would not all have the same lengths. Avalanche shape collapse is only possible if the system in question has scale-free properties in many domains, and this is empirically found to occur only when near a critical phase transition point.

By scale-free, we mean that some relationships between numbers will be the same across scales. To illustrate, consider the Gutenberg-Richter law for earthquakes. Here, there is a power law relationship between the frequency of an earthquake occurring and its energy. An earthquake with a magnitude of 7 on the Richter scale has 10 times the energy of a Richter scale 6 earthquake; it also occurs only 1/10 as often as Richter scale 6 earthquake. Thus, there is an inverse relationship, by powers of 10, between earthquake magnitude and frequency per year. This relationship occurs again between Richter scale 3 earthquakes and Richter scale 2 earthquakes. The former have 10 times the energy but occur 1/10 as often. For any pair of adjacent magnitudes, this type of relationship will apply–at the smallest scales and also at the largest scales. This is why power laws are often called scale-free. When a system is operating very close to a critical point, its activity is expected to be scale free. By this, we mean that many variables of the system will follow power law relationships. With the neuronal avalanches we discussed previously, this was shown in the distribution of avalanche sizes and in the distribution of avalanche lengths. Recall also Figure 1E, where there is a relationship between avalanche size and duration. Without this relationship, avalanche shape collapse would not be possible. Shape collapse is thus an indicator that the network is operating near a critical point. The existence of a universal scaling function, in our case the inverted parabola, is evidence that even the shapes of things replicate across different scales. Because a parametric description of this shape would require not just a single number, but several, it is considered by physicists to be an excellent indicator that a system is near a critical point (Spasojević et al., 1996; Papanikolaou et al., 2011). Power laws, in contrast, are typically described by only one number, their slope.

Let us now discuss another indicator of proximity to a critical point. Each of the power laws we mentioned has its own slope, given by its exponent: τ for avalanche size, α for avalanche duration, and γ for avalanche size vs. duration. The values of these exponents cannot be arbitrary if everything is scale-free; they must interlock in just the right proportions if they are to describe avalanches whose sizes and durations are all fractal copies of each other. By simple reasoning, described in Scarpetta et al. (2018), one can show that they must be related by this exponent relation equation:


$$\frac{\alpha -1}{\tau -1}=\gamma$$

This then is another signature of a neural network operating near a critical point–the exponents obtained from empirical data must satisfy this equation within some statistical limits (Ma et al., 2019). This relationship has been adopted by experimenters using cortical slice cultures (Friedman et al., 2012), zebrafish (Ponce-Alvarez et al., 2018), turtles (Shew et al., 2015), mice (Fontenele et al., 2019), rats (Ma et al., 2019), monkeys (Miller et al., 2019), and humans (Arviv et al., 2015) to assess closeness to a critical point. There is currently much work exploring why so many data sets follow this relation (Carvalho et al., 2020; Fosque et al., 2021; Mariani et al., 2021).

Criticality can also be suggested by long-range temporal correlations, and these have often been reported in neuronal data (Linkenkaer-Hansen et al., 2001; Lombardi et al., 2012, 2014, 2021; Meisel et al., 2017a,b). As we mentioned earlier, when a system is brought to the critical point, both spatial and temporal correlations can become scale-invariant.

**FIGURE 2:**
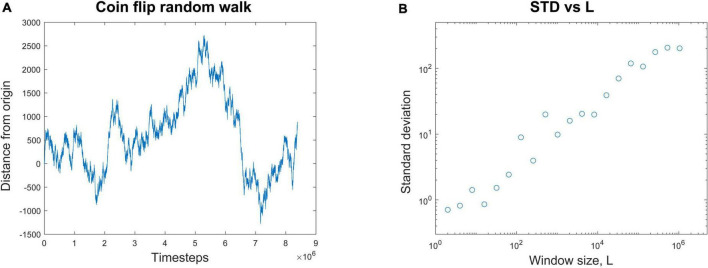
Calculating the Hurst exponent from a random walk. **(A)** A random walk process is started at the origin. At each time step, it randomly moves either forward (+1) or backward (–1) by one step. The position of the walker is plotted against the number of time steps. **(B)** The average standard deviation of the random walk is plotted for window lengths, *L*, of many different sizes. If *L* = 8, for example, the entire random walk is broken up into segments of eight time steps each and the average standard deviation from them all is calculated. When the average standard deviation for each window size is plotted against window size, a nearly linear relationship is revealed in these log-log coordinates. The Hurst exponent, *H*, is the slope of the best fit line through these data. In this case, *H* = 0.47. This linear relationship is evidence of scaling; when *H* > 0.5, it is evidence of long-range temporal correlations (LRTCs), often found in systems operating near the critical point.

A common way to quantify temporal correlations is through the Hurst exponent, *H*. This describes how the standard deviation scales with the duration of the data. See Hardstone et al. (2012) and Ihlen (2012) for excellent tutorial reviews with software. For example, consider a random walker on the number line whose position, x, is known over time (Figure 2A). It starts at the origin and takes either a step forward (+1) or backward (−1) with equal probability.

To illustrate how to calculate *H*, let’s consider a simulation of this process. We observe that after *t* = 131,072 time steps, the standard deviation is measured to be 70.26. Next, we expand the recording length by a factor of *L* = 8. By how much will the standard deviation, *STD*(x), increase? We want to know if the standard deviation is somehow related to the duration of the recording in a scale-free manner. In other words, the standard deviation should scale with the duration by some exponent. To continue our example, we observe that over *L* × 131,072 = 1,048,576 time steps the standard deviation is now measured to be 202.13. We can relate these numbers through the equations below to find the scaling exponent *H*. Recall that in our example, *t* = 131,072, *L* = 8, and the standard deviation when *t* = 131,072 is just 70.26 (*STD*(131,072) = 70.26). We now want to find *H*:


$$\,S\,T\,D\,\left(L\,t\right)=L^{H}\,S\,T\,D\,\left(t\right)$$

$$S\,T\,D\,\left(8\times 131,072\right)=8^{H}\,S\,T\,D\,\left(131,072\right)$$

$$S\,T\,D\,\left(1,048,576\,\right)=8^{H}\,S\,T\,D\,\left(131,072\right)$$

$$\,202.13=8^{H}\,70.26$$

$$\,\frac{202.13}{70.26}=8^{H}$$

$$\,\log_{8}\left(2.88\right)=\log_{8}\left(8^{H}\right)$$

⁢0.51=H

Here the Hurst exponent is approximately 0.5, which matches the analytic results for a random walk (Tapiero and Vallois, 1996). For any window of length *L*, the standard deviation of the random walk will be *L^H^*. If we plot the standard deviation for each window length *L* against the window length, we can get several data points (Figure 2B). The slope of the best fit line through these points will give an estimate of the Hurst exponent *H*; in this case it is *H* = 0.47. This is then a scale-free relationship, like what we saw with avalanche shapes, where the duration and height of the avalanches had the same relationship across all scales.

The Hurst exponent can also tell us things about long-range temporal correlations. In the case of the random walk, there is no temporal memory. This means that each step taken is independent of all the previous steps. For memoryless processes like these, the Hurst exponent is known to be about 0.5 (Hu et al., 2013).

But there are processes where some temporal memory is present. What happens if each successive step is influenced by previous steps? For example, in a correlated random walk, we could make it such that a step in one direction would slightly increase the odds of drawing another step in the same direction. This would cause the random walker to move away from the origin more rapidly than in the balanced, uncorrelated situation. In this case, the standard deviation would grow more quickly with the recording duration and so the Hurst exponent would be greater than 0.5. Conversely, if we made it such that a step in one direction would slightly decrease the odds of drawing another step in the same direction (anticorrelated), the walker would remain closer to the origin and the Hurst exponent would be less than 0.5 (Hu et al., 2013). In EEG data from humans, *H* has been reported to be in the range of 0.55 ≤ *H* ≤ 0.85 over several frequency bands (Colombo et al., 2016). These data show that neuronal processes near the critical point are not memoryless–they are correlated. Experiments have shown that temporal correlations in systems near the critical point do not decay as exponentials but as power laws (Linkenkaer-Hansen et al., 2001). Thus, long-range temporal correlations are another signature of criticality that have been consistently reported in neural data.

Continuing with signatures of being near the critical point, it is important to mention the recent advancements made by Wilting and Priesemann in estimating the branching ratio σ under conditions of sparse data sampling (Wilting and Priesemann, 2018). Recall that σ should be very close to one when the network is near a critical point. When they applied their method to data sets of spiking activity recorded *in vivo* from monkeys (*n* = 8), cats (*n* = 1), and rats (*n* = 5), they found the average value to be σ = 0.9875 ± 0.0105 (Wilting and Priesemann, 2019). In our own data from hundreds of measurements taken from networks of primary cultured neurons, we find the mode of the branching ratio to be σ = 0.98 (Timme et al., 2016). While there is still some discussion as to whether the networks are exactly at a critical point or slightly below it, there is now growing consensus they are very near it. Being near a critical point to optimize information processing would still be consistent with the criticality hypothesis.

Taken together, these advancements show that the field has tools beyond power laws to assess proximity to a critical point. When the control parameter in a tunable system is moved, we can now tell with confidence when the system is near criticality. These criteria for assessing criticality will be useful later when we examine systems based on random walks.

## Objection one: The Brunel network model is not critical and does not show a peak in information processing at the phase transition. Reply: When properly tuned and stimulated, the Brunel model shows a critical phase transition and a peak in mutual information

We will now consider the first objection, raised by Jonathan Touboul and Alain Destexhe in their paper entitled “Power law statistics and universal scaling in the absence of criticality,” (Touboul and Destexhe, 2017). There, they claim that the well-known Brunel model (Brunel, 2000) of spiking cortical networks does not show critical dynamics. Further, they claim that this model does not show a peak in information capacity. As the model is expected to represent cortical network dynamics, these results would seem to refute the criticality hypothesis.

**FIGURE 3:**
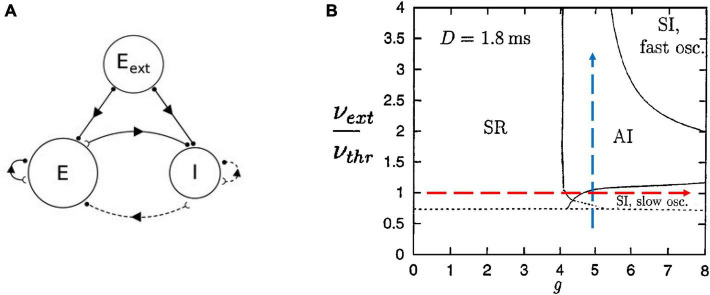
The Brunel model and its phase space. **(A)** Schematic of the Brunel model. It consists of an excitatory population of neurons (E), an inhibitory population (I), and a source of external excitatory drive (E_ext_). Excitatory connections are given by solid lines and inhibitory connections are given by dashed lines. **(B)** The phase space of the model is plotted as a function of two parameters, the ratio of external drive to the drive needed to exceed threshold (ν_ext_/ν_thr_), and the relative strength of inhibitory connections (g). There are four main regions, or phases: SR for synchronous regular; AI for asynchronous irregular; SI with slow oscillations; SI with fast oscillations. The dashed arrows represent the types of paths we will take in parameter space to explore the model. We will increase inhibition while keeping external drive fixed (red horizontal arrow) and we will increase external drive while keeping inhibition fixed (blue vertical arrow). Delays between neurons were 1.8 ms (*D* = 1.8 ms). Panel **(A)** is adapted from Nordlie et al. (2009); panel **(B)** is adapted from Brunel (2000).

We can begin by describing the Brunel model (Brunel, 2000). Briefly, it consists of leaky integrate-and-fire neurons that are sparsely connected so that 10% of all possible connections are present. It contains 80% excitatory neurons and 20% inhibitory neurons; there is also an external input to simulate thalamic drive (Figure 3A). Nicolas Brunel showed that by tuning the parameters of this model, like the relative strength of inhibition compared to excitation, he could cause it to display different phases of activity commonly reported in experimental studies of cortical networks (Figure 3B). For example, the synchronous regular (SR) phase was characterized by neurons firing synchronously in a rhythmic, or regular, manner reminiscent of cortical oscillations. Recordings of cortical neurons *in vivo* have been typically thought to fire with AI activity, where neurons do not tend to fire at the same time and there is no pronounced rhythm, while those *in vitro* have been thought to fire with SI patterns characterized by simultaneous firings but not at regular intervals. However, a recent report of *in vivo* activity in awake behaving rodents has shown that over several hours activity often switches between AI and SI phases, with signatures of criticality found between them (Fontenele et al., 2019). The Brunel model can capture all these activity phases.

**FIGURE 4:**
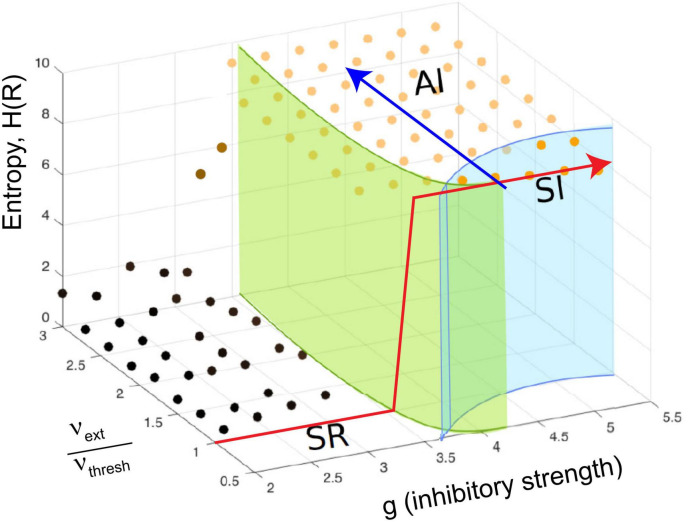
The continuously driven Brunel model does not show a peak in response entropy. The diagram shows the phase space of the model as a function of two parameters: g, the relative strength of inhibition, and the ratio of external drive to drive required to exceed threshold, ν_ext_/ν_thresh_, similar to what was shown previously in Figure 3B. The *z*-axis gives the entropy of the activity produced by the network in response to this drive, H(R). Each dot shows a location in parameter space that was sampled with the model by Touboul and Destexhe (2017). As described earlier, there are multiple phases: SR, synchronous regular; AI, asynchronous irregular; SI, synchronous irregular. One phase transition could occur at the boundary between regular (SR) and irregular (AI/SI) activity. For a given ratio of ν_ext_/ν_thresh_, the entropy increases in a step-like manner as g is increased through this transition. This is shown by the red line, which jumps upward near *g* = 3.5 and stays elevated. It does not drop back down as expected from the critical brain hypothesis. Another phase transition could occur as the model is moved from the SI phase to the AI phase at a constant value of g (blue line). Along either path, the response entropy does not show a peak as the model transitions from one phase to another. The model here is being constantly driven by random external input whose strength is equal to the strength of internal feedback connections within the network. Adapted from Touboul and Destexhe (2017).

In Touboul and Destexhe’s implementation, they made the external random drive equal in strength to the drive from excitatory neurons within the model. With this, they showed that as the model was tuned from the SR phase to the AI and SI phases by increasing the relative strength of inhibition, there was a jump in the entropy of the network activity, also known as the information capacity. The information capacity did not drop back down after the transition; rather it stayed high throughout the AI and SI phases (Figure 4, red arrow). They did not observe a peak near the transition to the SI phase, where they found power law distributions. Yet this should be expected by the criticality hypothesis. In addition, as they increased the external drive by raising the ratio ν_ext_/ν_thresh_ for a fixed value of g, they did not observe a peak in response entropy either (Figure 4, blue arrow). They claimed that the lack of a peak in the information capacity argued against the critical brain hypothesis, which would predict a peak near a phase transition. They stated “…we observe no difference between entropy levels in the SI or AI states, Therefore, we conclude that the maximality of entropy is not necessarily related to the emergence of power-law statistics” (Touboul and Destexhe, 2017, pages 7–8).

**FIGURE 5:**
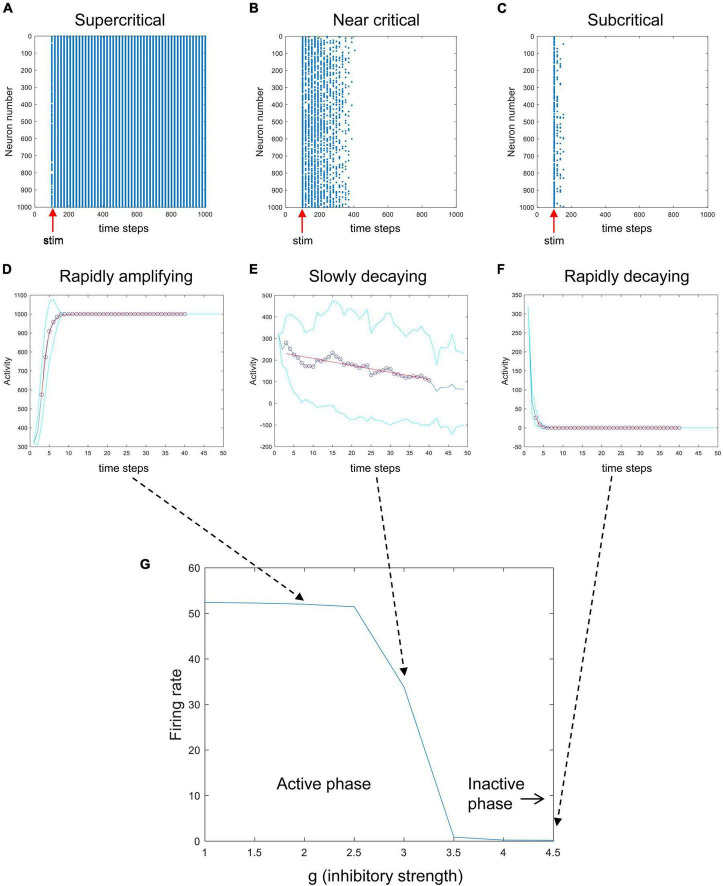
A phase transition in the Brunel model. Here, the random external drive was turned off and stimulation was delivered once at a prescribed time. The top row shows raster plots of activity produced by the Brunel model when it was stimulated once at the 100th time step (red arrows). Stimulation consisted of randomly activating 320 out of 1,000 neurons. Each spike is given by a blue dot. **(A)** When the inhibitory connection strength, g, was low, activity rapidly increased. **(B)** When g was at an intermediate value, activity died out slowly. **(C)** When g was large, activity was quickly damped. The middle row shows the average number of neurons activated after stimulation for the three conditions **(D–F)**. Each curve shows an average of 30 trials. Cyan curves show one standard deviation. Exponential curves in red were fit to time steps 3 through 40, shown in circles. Note that while conditions **(D,F)** have opposite directions of growth, they both have short time constants (sharply bending curves). Condition **(E)**, in contrast, has a long time constant (gradually bending curve). **(G)** The average firing rate for the model is plotted against different values of g, showing a clear transition from an active phase to an inactive phase. Matlab code for producing all these plots is given in the Supplementary material.

To investigate these issues, I modified the Matlab code used to simulate the Brunel model that was freely provided by Destexhe and Touboul in their most recent paper on this subject (Destexhe and Touboul, 2021). I explored the model under more controlled conditions, where I could deliver stimulation pulses and observe the response of the network without random background activity. I brought the external drive to zero and, for example, activated 320 randomly chosen neurons (out of 1,000) only at one given time step. The results of these experiments are shown in Figure 5. When the parameter g, which controls the relative strength of inhibition, is low, then activity is quickly amplified (Figures 5A,D). When g is at an intermediate value, stimulation produces very slowly decaying activity (Figures 5B,E). When g is large, strong inhibition quickly dampens activity from the stimulus (Figures 5C,F). This shows that the network can be tuned from an active, amplifying phase to an inactive, dampening phase as g is increased. Here, g serves as the control parameter for tuning the network while the average firing rate serves as the order parameter giving the phase of the system. This path through phase space, changing g while keeping the amount of external drive fixed, is like the path shown by the red arrow in Figures 3B, 4. The phase plot shown in Figure 5G looks just like what we should expect for a system with a critical phase transition.

**FIGURE 6:**
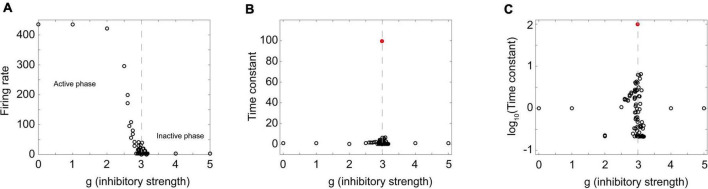
A sharp peak in the time constant near the phase transition point. **(A)** The firing rate in a Brunel model with 8,000 neurons is plotted as a function of the inhibitory strength g. The model was stimulated by randomly activating 20% of the neurons; the number of spikes divided by the total number of time steps was taken to be the firing rate. Note active phase on the left and inactive phase on the right. Dashed gray line denotes approximate border between them. **(B)** Time constant for exponential decay of activity after stimulation shows a sharp peak near the phase transition. Peak value of time constant occurred at *g* = 2.99 and is indicated by the red circle. Dashed gray line again drawn at transition. **(C)** Same plot as shown in panel **(B)**, but with the time constant measured in log scale, showing transition region in more detail. A total of 93 different values of g were probed. Probing was densest near the transition region; each probe consisted of 30 stimulations of the network. Exponential curves were fit to each stimulation and the average time constant for the 30 stimulations is shown as a single circle. Matlab code for producing all these plots is given in the Supplementary material.

If this really is a critical phase transition, then we should also expect to see peaks in some functions near the phase transition point. To examine this, I extracted time constants from a Brunel model with 8,000 neurons with activity curves like those shown in Figure 5D through Figure 5F. As the relative strength of inhibition was increased, the model showed a transition from an active phase to an inactive phase (Figure 6A). When the 8,000 neuron network was near the phase transition point (*g* = 3) activity decayed quite slowly. But away from the phase transition point this was not the case. In the amplified network, activity quickly saturated so the time constant was short; in the dampened network, activity quickly died so the time constant was also short. Plotting the time constants against the control parameter revealed a sharp peak near the phase transition (Figures 6B,C). This showed that temporal correlations were maximized, just as we would expect in a critical system.

**FIGURE 7:**
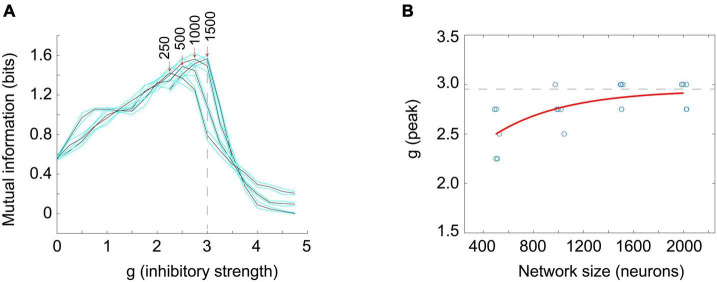
The peak in the mutual information coincides with the peak in the time constant. **(A)** Mutual information between the response and the stimulus was measured in Brunel models of different sizes (250, 500, 1,000, and 1,500 neurons shown) as the inhibitory strength, g, was varied. Stimuli consisted of eight different numbers of neurons (e.g., 0, 125, 250, 375, … for *N* = 1,000 neuron model) randomly activated at one time; the average number of neurons active at time steps 3 through 5 after the stimulus was taken as the response. Black curves show mutual information for each model; cyan curves show one standard deviation. Five models of a given size were run 30 times each to produce each data point; more details are in the Supplementary material. Peak mutual information values for each model size are indicated by red arrows. Note that as model size increases, peaks become taller and move toward *g* = 3. Dashed vertical line is at *g* = 3 for reference. **(B)** The value of g at which mutual information peaks is plotted against model sizes (*N* = 500, 1,000, 1,500, 2,000 shown). Blue circle tokens were jittered slightly to improve visibility. An exponential fit to these data gives an asymptotic value of *g* = 2.952, with 95% confidence bounds at 2.637 and 3.268. This is within experimental error of the peak in the time constant found for the Brunel model with 8,000 neurons (*g* = 2.99) shown in Figures 6B,C. This agreement of peak values in mutual information and time constant duration is what would be expected for a second order phase transition and supports the hypothesis that the Brunel model has a critical point. It also supports the criticality hypothesis. Matlab code for producing all these plots is given in the Supplementary material.

Next, I turned to examine the mutual information. Here, I stimulated a network at one time step with eight different numbers of randomly chosen neurons (thus giving 8 = 2^3^ or 3 bits of input entropy) and used the number of neurons active in the network as a measure of the response. As many readers may know, the mutual information can be written as a difference between two quantities, the entropy of the response, H(R), and the entropy of the response conditioned on the stimulus, H(R| S). Thus we have: MI(S;R) = H(R)–H(R| S). For mutual information to be high there should be a wide variety of responses, making H(R) large, and a narrow and reliable set of responses for each given stimulus, making H(R| S) low. The information capacity is merely the H(R) term and does not include the H(R| S) term. Note it is possible to have a high information capacity but low mutual information if H(R| S) is large. This would occur if the network had highly variable output and rarely gave the same response to a given stimulus. When Touboul and Destexhe measured the information capacity, they were only measuring H(R) and were not delivering stimuli. Thus, they did not measure information transmitted through the network. When the mutual information MI(S;R) was measured in the stimulated Brunel networks, I found a peak near *g* = 3, in agreement with the peak for the time constants (Figure 7). For smaller networks this peak occurred for lower values of g, but as network sizes were increased, the peak value of g asymptotically approached *g* = 2.952, in good agreement with the peak value of g for the time constant measurements (*g* = 2.99). These findings suggest that the Brunel model indeed has a phase transition and that information transmission is maximized there. This all agrees with the criticality hypothesis stated earlier.

Given this result, one might wonder why Touboul and Destexhe reported only that there was no peak in information capacity. First, as we just explained, information capacity is not the same thing as mutual information. The criticality hypothesis predicts a peak in information transmission through a network when it is at a critical point. To assess this, one must measure mutual information, which is the difference between the information capacity, H(R), and the conditional entropy, H(R| S). Second, the Brunel model tested by Touboul and Destexhe was receiving external input that had a total synaptic weight equal to the weight of all the excitatory neuron synapses within the network. This arrangement made the network activity very dependent on the random external drive and made the internal network dynamics more difficult to observe. Interestingly, experiments in cortical slice networks have shown that limited thalamic stimulation does not seem to disrupt ongoing cortical network activity (MacLean et al., 2005). However, more recent work has shown that an intrinsically critical network will be pushed away from the critical point as external drive is increased (Fosque et al., 2021). This makes it reasonable to explore how the network functions under conditions of reduced external drive. To see how influential strong random drive can be, let us move on to the second objection.

## Objection two: Very simple unconnected models can show signatures of criticality when randomly driven. Reply: Such models have many of these signatures, but the signatures largely arise from the noise source itself. Such models also fail to account for experiments showing neuronal criticality: (1) Processes information, (2) depends on connections between neurons, and (3) produces long-range temporal correlations

**FIGURE 8:**
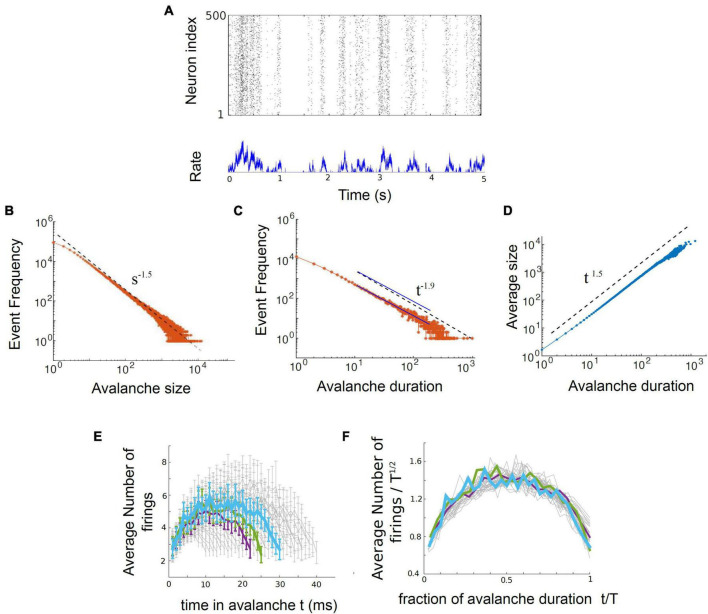
The output of the Ornstein-Uhlenbeck model implemented by Touboul and Destexhe (2017). **(A)** A raster plot of spiking activity. Neuron number is on the *y*-axis, and time is on the *x*-axis. Each dot represents a spike from a model neuron. The summed activity of all the neurons in each time bin is plotted below to show how firing rate changes with time. Here it is in the synchronous irregular (SI) phase. **(B)** Distribution of avalanche sizes for the model approximately follows a power law with exponent –1.5. **(C)** Distribution of avalanche durations approximately follows a power law with exponent –1.9. The two blue lines are parallel and are shown as an example of a slope that would be slightly shallower than the one found by Touboul and Destexhe (2017). **(D)** Average avalanche size for a given duration, plotted against avalanche duration, approximately follows a power law with exponent 1.5. **(E)** Average avalanche shapes, for durations of 10–40 time steps (ms) of the model. **(F)** Avalanche shapes collapse well for exponent of γ = 1.5, as expected. Figure adapted from Touboul and Destexhe (2017).

In their 2017 paper, entitled “Power law statistics and universal scaling in the absence of criticality,” Touboul and Destexhe continued their computational experiments by exploring the behavior of an ensemble of neurons without internal connections that received strong, correlated, randomly varying external drive. They first showed that this model could produce power law distributions of avalanches that satisfied avalanche shape collapse (Figure 8; Touboul and Destexhe, 2017), and later showed that it could satisfy the exponent relation (Destexhe and Touboul, 2021). This model suggests that the signatures of criticality observed in neuronal experiments may be produced by a simple process, like a random walk, that is passed through a threshold. In other words, the criticality that has been claimed to exist in living neuronal networks may not be the result of collective interactions among neurons. If true, this would contradict the criticality hypothesis mentioned earlier, which claims that optimal information processing is emergent near the critical point and depends on interactions between neurons.

My response will consist of five parts. (A) First, we will cover some details of their unconnected model and its external noise source. (B) Second, to understand the contributions of external noise, we will examine how the behavior of the Brunel model changes when it is driven by *uncorrelated* random noise. (C) Third, we will examine how the behavior of the Brunel model changes when it is driven by *correlated* random noise. These two types of drive produce materially different behaviors, although both degrade information processing. (D) Fourth, we will examine the properties of the correlated noise itself and show that it can be tuned to produce power laws and even avalanche shape collapse, something that cannot be shown for uncorrelated noise. This will explain how even a network with no internal connections can show signatures of criticality. (E) Fifth, we will draw three distinctions between the signatures of criticality produced by correlated noise and those produced by a connected network of neurons. Connected networks transmit information well, have emergent criticality that depends on connections, and show long-range temporal correlations. In contrast, disconnected networks transmit information poorly if at all, do not display emergent criticality and show no long-range temporal correlations. We will show that the experimental data are consistent with connected network models but not with disconnected network models.

### Understanding the disconnected model

Let us now turn to the model. It consists of an ensemble of neurons with no internal synaptic connections that is driven by external noise. This noise source is an Ornstein-Uhlenbeck process that we will explain more below. The model output can be seen in Figure 8, taken from Touboul and Destexhe (2017). There, three power law plots are shown for avalanche size, duration, and size vs. duration. In addition, the average avalanche shapes show excellent collapse. While the exponents given from these power laws (τ = 1.5, α = 1.9, γ = 1.5) do not closely satisfy the exponent relation [(1.9–1)/(1.5–1) = 1.8 ≠ 1.5], they are not too far off. A slightly shallower slope of α = 1.75 for the avalanche duration plot, for example, would cause the exponent relation to be satisfied.

**FIGURE 9:**
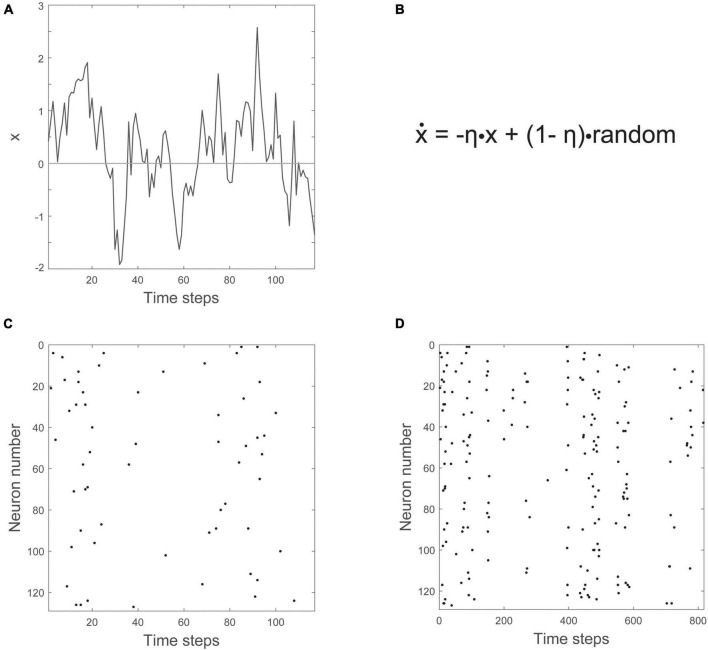
The Ornstein-Uhlenbeck model. **(A)** The variable x moves up and down across a threshold over time. The threshold is given by the horizontal line at zero. **(B)** The equation governing how x changes (ẋ) has negative feedback (-ηx) and a random noise term (+(1-η)⋅random). **(C)** Number of neurons firing is proportional to how far x is above threshold. **(D)** A zoomed out view of the raster plot, showing synchronous irregular (SI) activity.

How does the Ornstein-Uhlenbeck process drive the population of neurons? It has a single variable, x, that zigs and zags across a threshold, as shown in Figure 9A. The equation governing the behavior of x is given in Figure 9B, and has two parts, one of which is random. The other part has the effect of constraining, or counteracting, the randomness. To turn the movements of this single variable into something that could represent a population of neurons, a threshold is introduced. Here, to prevent inaccuracies that can arise from setting the threshold too high (Villegas et al., 2019), we will set the threshold at zero. Whenever x crosses above the time axis, we can say that neurons become active. Whenever it is below the axis, there is no activity. The number of neurons activated is proportional to the height of x. For example, shortly before 20 time steps, x has a value of about 2. When multiplied by a proportionality constant of 3, this would mean that six neurons should be active at that time. Six neurons are randomly chosen out of the population and made active. When this is done for 128 neurons over about 800 time steps, you get the raster of activity shown in Figures 9C,D.

Clearly a very important part of this model is the random drive that activates the disconnected population. To better understand the role of random drive, let us return to the Brunel model. Recall that it can be tuned to a critical point when external drive is limited. Now we will examine the Brunel model dynamics when it is continually driven by two types of noise: uncorrelated and correlated. This will position us to better understand how the Ornstein-Uhlenbeck model can generate signatures of criticality, and how those signatures fail to match what is observed in experiments with living neurons.

### Brunel model driven by uncorrelated random noise

**FIGURE 10:**
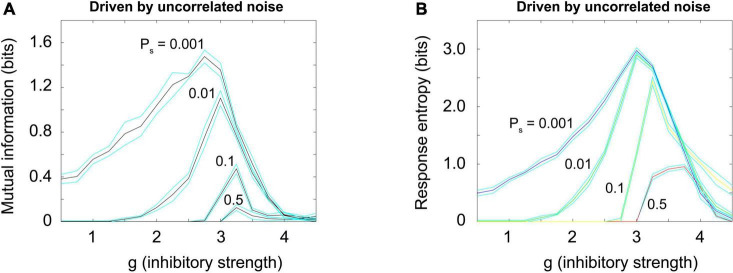
Random uncorrelated noise reduces mutual information and response entropy. **(A)** Mutual information was plotted for a Brunel model with 1,000 neurons, using the same procedures described previously, but now with varying amounts of externally generated uncorrelated noise. Noise was simulated by giving each neuron an independent probability of spontaneously firing, P_*s*_, that varied (0.001, 0.01, 0.1, 0.5). This noise is uncorrelated because the activity in the randomly driven neurons was independent. Note that as uncorrelated noise is increased, the peak in mutual information declines. **(B)** Response entropy also declines as uncorrelated noise is increased. These results are consistent with the principle of quasicriticality (Williams-Garcia et al., 2014; Fosque et al., 2021). Matlab code for producing these plots is given in the Supplementary material.

Figure 10A shows how the mutual information curves from the Brunel model (shown previously in Figure 7A) change when uncorrelated noise is added, as Brunel originally proposed (Brunel, 2000). Increased noise decreases the mutual information; it also reduces the response entropy, as shown in Figure 10B. These results are consistent with the principle of quasicriticality (Williams-Garcia et al., 2014; Girardi-Schappo et al., 2020; Fosque et al., 2021), which describes how external drive will affect information processing functions in networks near the critical point.

Note also that by increasing external drive, we are moving through phase space along the path of the blue arrow shown in Figures 3B, 4. But uncorrelated noise is not the only type of noise that we need to consider along this path. There is much research investigating the effects of correlated noise on neural networks (Lee et al., 1998; Cohen and Kohn, 2011). Let us explore this next.

### Brunel model driven by correlated random noise

**FIGURE 11:**
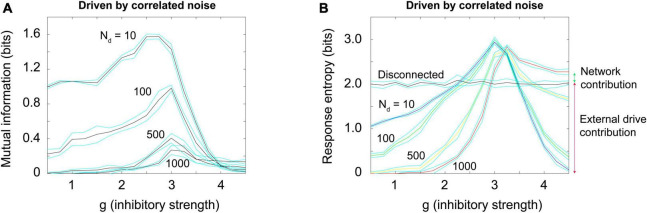
Random correlated noise reduces mutual information but *can increase* response entropy. **(A)** Mutual information was plotted for a Brunel model with 1,000 neurons, using the same procedures described previously, but now with varying amounts of externally generated correlated noise. Correlated noise was added by simultaneously driving varying numbers of neurons (*N*_*d*_ = 10, 100, 500, 1,000) with a rectified coin flip process (details described in text and in Supplementary material). This type of drive was also used by Touboul and Destexhe (2017) and Destexhe and Touboul (2021). Note that as this noise is increased, the peak in mutual information declines. **(B)** Response entropy, however, remains high near the peak (close to *g* = 3) for all numbers of neurons driven, and in the case of 1,000 neurons driven, it remains high even as g is increased beyond 3. This is consistent with the findings of Touboul and Destexhe (2017), who called the response entropy the “information capacity.” Note however that the mutual information, shown in panel **(A)**, does decline as external drive increases, consistent with the critical brain hypothesis. Note further that the high response entropy is largely the result of external drive and not the network itself: The horizontal black curve shows the response entropy produced by a network with no internal connections, only receiving external drive. This curve is slightly below that produced by the Brunel model with its default setting of 10% connectivity (red curve). The difference between these is shown by the small green arrow to the right of the plot; the response entropy produced by the disconnected network is shown by the taller red arrow to the right. There is about an 11% difference between them, indicating that 89% of the response entropy can be accounted for by the external drive alone. Matlab code for producing these plots is given in the Supplementary material.

To produce a correlated noise source, I followed one of the models used by Touboul and Destexhe (2017) and drove the network with the output of a rectified coin flip. I will explain this mechanism more later, but for now it is enough to state that it is conceptually similar to the Ornstein-Uhlenbeck process mentioned earlier. The total number of neurons that could be activated in a 1,000 neuron model was varied among these values: 10, 100, 500, and 1,000. By increasing the number of neurons that could be activated, we could increase the relative strength of the external drive. Again, this would be moving along the blue arrow in phase space, as shown in Figures 3B, 4. When correlated noise like this is added to the Brunel model, we also see a decline in mutual information (Figure 11A). This is expected, as random background unrelated to the stimulus will alter the input patterns to the network, making the response variability go up. Interestingly, strong correlated noise does not always reduce the response entropy (or information capacity), in contrast to what we observed when the model was driven by uncorrelated noise. This can be seen in Figure 11B by following the red curve produced by the *N* = 1,000 condition. Notice that this curve does not drop back down to the axis as the inhibitory strength g is increased beyond 3. Rather, it remains high, and is roughly 2.5 bits when *g* = 4. This is consistent with the findings reported by Touboul and Destexhe (2017) who noted that there was not a peak in the information capacity as the model was moved across a phase transition. As we pointed out earlier, though, there is a peak in the mutual information (Figure 11A), as predicted by the criticality hypothesis. Thus, mutual information does show a peak, even though information capacity does not clearly show one for high values of g.

Let us now explore the cause of this elevated response entropy. What will it look like if we disconnect the neurons from each other in the Brunel model, thus imitating what was done in the Ornstein-Uhlenbeck model used by Touboul and Destexhe (2017)? As shown in Figure 11B by the black curve, a disconnected model has nearly the same amount of response entropy as that found in a connected model (red curve). The difference between the curves is indicated by the small green arrow to the right of Figure 11B. Compare this to the red arrow, the response entropy produced by the connected model when 1,000 neurons are driven by correlated noise. From this we can conclude that nearly 90% of the response entropy is caused by the external drive, and only about 10% of it is caused by interactions among the neurons within the network.

Because this external drive is so dominant, it deserves further scrutiny. What are the statistical properties of a rectified coin flip? We will turn to this in the next section.

### Correlated random noise can show many signatures of a critical process

**FIGURE 12:**
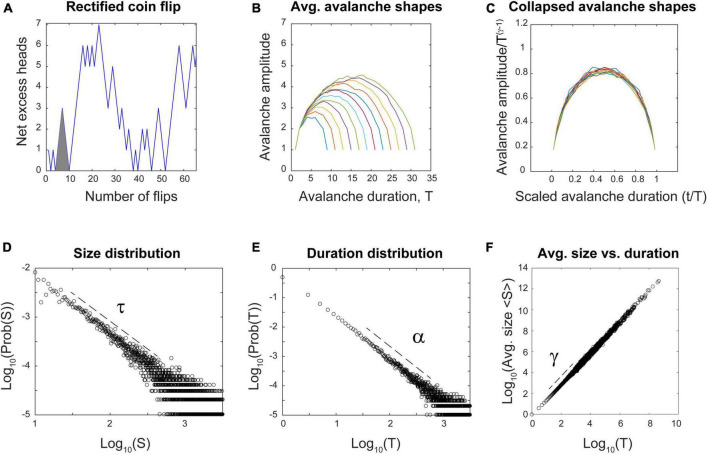
The reflected coin flip satisfies multiple signatures of criticality without any connections. **(A)** The reflected coin flip steps upward in y every time a head is flipped and downward for every tail. All negative excursions are reflected to produce only positive avalanches. Here, only 64 flips are shown; simulation had 2 billion flips. The gray triangle highlights an example run of six flips mentioned in the text. **(B)** Average avalanche shapes with durations from 8 to 32 are shown. **(C)** Average avalanche shapes are fractal copies of each other and show good collapse when rescaled using the exponent γ. **(D)** Avalanche size distribution followed a power law with exponent τ = 1.32. Distribution was significantly better fit by truncated power law than by other distributions (*p* = 0.786). **(E)** Avalanche duration distribution followed a power law with exponent α = 1.49. Distribution was significantly better fit by truncated power law than by other distributions (*p* = 0.402). **(F)** Average avalanche sizes plotted against durations follows a power law with exponent γ = 1.50. Using all three exponents, the error in fitting the exponent relation was 2.9%. Code for producing this simulation can be found in the Supplementary material. Dashed lines show approximate regions over which power laws were fit.

I will now explain the rectified coin flip process in more detail. We are all familiar with flipping a fair coin that has equal probability of landing heads (H) or tails (T). If we take this process and map it onto the number line, we could take one step forward (+1) for each head and one step backward (−1) for each tail. An example “avalanche” here could be a run like this: (H, H, H, T, T, T), as shown in Figure 12A (gray triangle). The process starts at the origin and returns there after an equal number of heads and tails have been flipped. The duration of this avalanche would be six, as there were six flips. The height of the avalanche at any given time is determined by the net excess of heads. In this case, it would be: (1, 2, 3, 2, 1, 0). The size of the avalanche would just be the sum of these numbers: 9. Let us introduce one last condition on this process–we will rectify it so that excursions from the origin that go negative will be made positive. This way, only the absolute value of avalanches will be considered. Now, what are the statistics of this process? Can it produce signatures of criticality?

In fact, it can. Analytic work has shown that the distribution of first return times to the origin of a fair coin flip follow a power law distribution (Kostinski and Amir, 2016). To probe this further, I simulated two billion coin flips and then plotted the resulting distributions, as shown in Figures 12D–F. They are all significantly better fit by power laws than by other distributions. Moreover, the exponents from these power laws satisfy the exponent relation, within an error of 2.9%. The exponent γ can be used to perform avalanche shape collapse, as shown in Figure 12C. All of these signatures of criticality are clearly satisfied.

In addition, the process is tunable: These signatures appear at the critical point, when the coin is exactly fair. They will disappear when the coin is biased to produce more heads than tails. These biased conditions reveal the two phases–one where heads occur most often and another where tails occur most often. Perfect symmetry occurs only when the coin is exactly fair, and the signatures of criticality appear right at the point where this symmetry is about to be broken, at the transition between phases.

However, the coin flip does not show long-range temporal correlations as measured by the Hurst exponent: *H* ≈ 0.5, indicating a memoryless process as we mentioned earlier. Still, many of the signatures of criticality we highlighted can be fulfilled by the coin flip.

This result suggests that a neural network model without internal connections and driven by an external source like a rectified coin flip could show some signatures of criticality. But these signatures would largely reflect the statistics of the noise source and not the network itself. To explore this situation further, we will next describe the differences between emergent and non-emergent criticality.

### Emergent vs. non-emergent criticality

To adequately address this situation, it is necessary to first explain a key difference between the type of criticality we saw in the Brunel model and the signatures of criticality we saw in the coin flip model. This will require a short digression into degrees of freedom.

In describing any system, it is important to mention how many degrees of freedom it has. Briefly, the *degrees of freedom* are the number of parameters that would be needed to accurately specify the system. For example, in the coin flip model there was only one degree of freedom, given by the probability of getting heads. This could be for example *p* = 0.500 in a fair coin, or *p* = 0.501 in a biased coin. In the Brunel network model, the parameters included not only the relative strength of inhibition (g) and the relative frequency of external drive (ν_ext_/ν_thresh_), but also the list of all the connections made between the neurons. This list would include at least 100,000 more parameters (10% connection density × 1,000 neurons × 1,000 neurons = 100,000 connections). These examples illustrate the difference between systems with few degrees of freedom and those with many.

**FIGURE 13:**
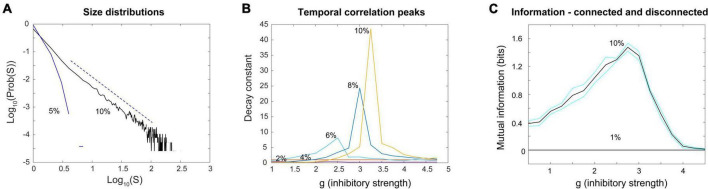
Power laws, peak decay constants and peak mutual information depend on connections between neurons in the Brunel model. **(A)** Avalanche size distributions deviate from power law form when connectivity between neurons is reduced. Black line shows power law distribution from the Brunel model when connectivity is set to the sparse default of 10%. It was statistically more similar to a truncated power law than other distributions (*p* = 0.238). Dashed line shows region over which power law scaling was found. Blue line shows downwardly curved distribution produced when connectivity is reduced to 5%. This was not statistically similar to a power law distribution (*p* = 0.076). **(B)** As connectivity is reduced from the default 10–2%, the time constant of temporal correlations drops and the peak near the phase transition point disappears. Note that as connectivity is increased, the peaks become taller and narrower, consistent with finite size effects observed in emergent criticality. Thus, temporal correlations in the Brunel model emerge from neuronal interactions near the critical point. **(C)** Mutual information shows a peak near *g* = 3 in the model when it has 10% connectivity, but this peak disappears completely when connectivity is reduced to 1%. Black lines are the average of five runs; cyan lines show one standard deviation. Each run of the model consisted of 30 networks constructed for each value of g. These figures demonstrate that criticality in the Brunel model emerges through the interactions among neurons. The code used for producing these figures is available in the Supplementary material.

The criticality hypothesis as stated earlier assumes that the brain is a system with many degrees of freedom and that criticality emerges there as a result of interactions between neurons. Criticality in systems with many degrees of freedom is most often studied with the tools of *statistical mechanics*. In the example of the piece of iron, the spins in the lattice must be influencing their nearest neighbors. In the example of a neural network model, the neurons must be capable of stimulating each other through synapses. In these types of systems, when the interactions are reduced in strength or cut, the signatures of criticality are diminished or disappear. Power laws are destroyed and peaks in mutual information or temporal correlations are flattened. A way to probe this type of criticality is by observing what happens in the system when the connections are manipulated. An example of this is given in Figure 11, where the density of connections in the Brunel model is reduced. When they are, the model no longer produces a power law of avalanche sizes (Figure 13A); it no longer has a sharp peak in the time constant at the phase transition (Figure 13B); it no longer transmits information through the network (Figure 13C). Signatures of criticality in the Brunel model clearly depend on interactions within the ensemble; this criticality is therefore emergent. Other investigators have also recently noted that emergent criticality can be distinguished from external effects by tracking the mutual information (Nicoletti and Busiello, 2021; Mariani et al., 2022). This approach should be very fruitful in future studies.

In contrast, systems with few degrees of freedom may not have any connections at all. Signatures of criticality can still exist in these systems but can arise over time as a single unit interacts with itself. Activity in these systems is typically studied from the perspective of *dynamical systems*. For example, consider a single unit whose dynamics are given by the branching ratio, σ. If a small perturbation to the system grows over time (σ > 1), it is chaotic. If a small perturbation shrinks over time (σ < 1), it is damped and stable. Only when a small perturbation is neither amplified nor damped but relatively preserved (σ = 1) is the system poised near the critical point. For activity to arise in these systems, they need to be driven, and this often comes from an external source of randomness. This external drive is sometimes called a *latent variable* (Schwab et al., 2014). When internal connections are cut in systems like these, it does not affect signatures of criticality. It would make no difference in the Ornstein-Uhlenbeck model proposed by Touboul and Destexhe because there are no connections to begin with.

Which type of criticality is observed in living neural networks? Fortunately, there are numerous experiments that have addressed this through the application of pharmacological agents that disrupt synaptic transmission. If the non-interacting models with few degrees of freedom are correct, then this should have no effect on signatures of criticality. But if criticality emerges through the interactions of neurons in systems with many degrees of freedom, then these manipulations should disrupt signatures of criticality.

**FIGURE 14:**
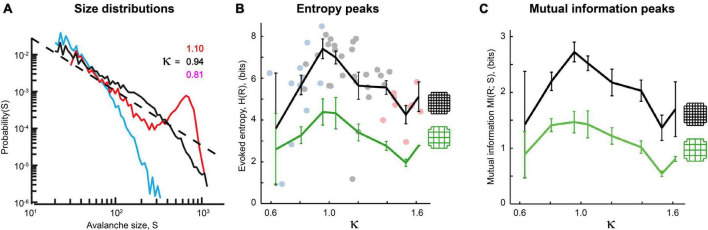
Power laws and peak information transmission depend on connections between neurons in living neural networks. **(A)** Avalanche size distributions recorded *in vivo* deviate from power law form when synaptic transmission is manipulated. Black line shows approximate power law distribution from an unmanipulated recording; dashed line has slope τ = −1.5 for reference. Blue line shows downwardly curved distribution caused by enhancement of inhibitory synaptic transmission (application of muscimol). Red line shows distribution when inhibitory synaptic transmission is disrupted (application of bicuculline). The value of κ parameterizes how close a given distribution is to an ideal power law (κ≈ 1 if nearly critical, κ > 1 if supercritical, κ < 1 if subcritical). Adapted from Gautam et al. (2015). **(B,C)** Pharmacological manipulations that disrupt excitatory synaptic transmission (application of AP5 and DNQX, blue dots) or inhibitory synaptic transmission (PTX, pink dots) reduce the evoked entropy **(B)** and the mutual information **(C)** between the stimulus and the response. Gray dots show network responses where no manipulations were applied. Adapted from Shew et al. (2011). Cortical slice cultures were placed on a 60-electrode array and stimulated electrically with 10 different amplitudes. The distribution of network responses to each stimulus provided H(R| S), while the distribution of network responses provided H(R). Mutual information is calculated as MI(R;S) = H(R)–H(R| S). Note that MI peaks at κ = 1. The black curve shows results produced by all 60 electrodes, while the green curve shows results produced by a coarse-grained approach where four neighboring electrodes are grouped together into a super-electrode. In both conditions, manipulations that disrupt synaptic connectivity reduce response entropy and mutual information. These figures demonstrate that the type of criticality in these networks depends on connections between neurons and is therefore emergent.

Let us now summarize results from a few of these experiments. Application of picrotoxin (PTX), a GABA_*A*_ antagonist that blocks inhibitory synaptic transmission, causes disruption of power laws in acute cortical slices (Beggs and Plenz, 2003). In this experiment, when picrotoxin was washed out the activity returned toward a power law distribution. Shew et al. (2009) showed that application of AP5 and DNQX, which together block excitatory synaptic transmission, disrupted power laws in organotypic cortical cultures. When the GABA_*A*_ antagonist bicuculline and the GABA_*A*_ agonist muscimol were applied *in vivo*, they tuned cortical activity away from a critical point and into the supercritical and subcritical phases, respectively (Gautam et al., 2015; Figure 14A). Similar manipulations also cause cortical slice networks to move away from peak information capacity (Figure 14B) and peak information transmission (Figure 14C). These manipulations of criticality are not confined to chemical synapses, though, as even the gap junction blocker heptanol disrupts the quality of avalanche shape collapse in zebrafish larvae (Ponce-Alvarez et al., 2018), again moving the neural network away from a critical point. In addition, these findings extend to human patients; antiepileptic drugs reduce cortical connectivity and produce subcritical avalanche size distributions as well as a reduction in long-range temporal correlations (Meisel, 2020).

Taken together, these results consistently demonstrate that neuronal criticality is an emergent phenomenon that depends on collective interactions between neurons. Thus, low-dimensional models without interactions are inadequate for capturing the type of criticality that occurs in the neural systems just mentioned.

Are there any cases from biology that support the models with few degrees of freedom? Several studies have noted that a randomly varying external drive, when applied to an ensemble of units, can produce apparent signatures of criticality (Schwab et al., 2014; Priesemann and Shriki, 2018), much like what we discussed with the Ornstein-Uhlenbeck and coin flip models. Swarming animals like starlings and insect midges have been shown to have spatial correlations that scale with the size of the swarm, suggesting criticality (Cavagna et al., 2010). However, when swarms of midges were isolated from external perturbations like wind and light, these correlations disappeared (van der Vaart et al., 2020); this has not yet been tried with starlings (release them into a domed stadium?). This result suggests that at least in the case of midges, signatures of criticality may not be intrinsic to the swarm itself but rather produced by an extrinsic source.

Let us finally return to the issue of long-range temporal correlations. Recall that in the coin flip process the Hurst exponent was *H* ≈ 0.5, indicating no temporal memory. Given that random walk models do not show emergent criticality or long-range temporal correlations, it is worth asking if there are models that do. Even the simple Brunel model showed a spike in its time constant, when the network was connected and tuned to the critical point (Figure 13B). It is known that a population of neurons modeled as a critical branching process can produce avalanche distributions that follow power laws, the exponent relation and show good shape collapse (Friedman et al., 2012). However, critical branching by itself can only produce short-range temporal correlations up to the length of the longest avalanche (Poil et al., 2008). But when neural network models include some type of homeostatic plasticity, over longer durations they can produce Hurst exponents up to *H* = 1, greater than the *H* ≈ 0.5 seen in random walks (Poil et al., 2012). Perhaps the addition of a simple temporal feedback term would allow random walk models to show long-range temporal correlations also, but they would still fail to show emergent criticality that depends on connections between neurons.

To conclude this section, random walk models fail to capture emergent criticality, they fail to support information processing, and they fail to exhibit long-range temporal correlations, all of which are observed in neural experiments. Because there are models that do capture these features (Poil et al., 2012; de Candia et al., 2021), random walk models should be discarded.

## Closing

The criticality hypothesis states that ensembles of neurons collectively interact to operate near a critical point. Near this point, they optimize multiple information processing functions simultaneously. The challenges to this hypothesis have taken several forms and have been met in different ways. Below is a summary of some recent challenges and how they have been addressed:

• An early objection was that many non-critical processes could produce power laws, so power laws alone were not sufficient to establish criticality. The field responded by developing additional ways to assess proximity to a critical point. These ways included the exponent relation, avalanche shape collapse, assessment of long-range temporal correlations and a better way of measuring the branching ratio. These methods are increasingly applied now and consistently show that many neural systems are operating near a critical point.
• A more recent objection is that even the well-known Brunel model of cortical networks does not show a peak in information capacity as it is tuned across a phase transition. In this paper, we saw that mutual information did show a peak, even if information capacity did not, when the Brunel model was decoupled from strong, correlated random drive. This result was consistent with the criticality hypothesis.
• Another recent objection is that even simple random walk processes like a coin flip can display signatures of criticality. This raises the possibility that signatures of criticality in living neural networks do not arise from collective interactions, but merely from external sources of randomness. While these simple models do produce many signatures of criticality and might be considered critical in some sense, they are unable to capture the type of criticality that emerges in neural networks through the interactions of many neurons. Because neuronal experiments show criticality produces a peak in mutual information, depends on synaptic transmission, and has long-range temporal correlations, these simple models are inadequate.

It is worth reviewing that in two of the objections, random noise played a key role. The peak in mutual information we observed in the Brunel model is suppressed in the presence of strong, external random drive (Figures 10A, 11A). The recent principle of quasicriticality notes that increased external drive will push an intrinsically critical network slightly away from the critical point (Williams-Garcia et al., 2014; Fosque et al., 2021). And paradoxically, if all we have is a random walk or a coin flip, we can get some signatures of criticality (Figure 12), except for a peak in mutual information and the long-range temporal correlations. This highlights that apparent criticality can arise in two different ways–either intrinsically, through interactions among many units in an ensemble, or extrinsically, through a driving process or latent variable like a random walk. How then can we decide what type of system we have if both show some signatures?

**FIGURE 15:**
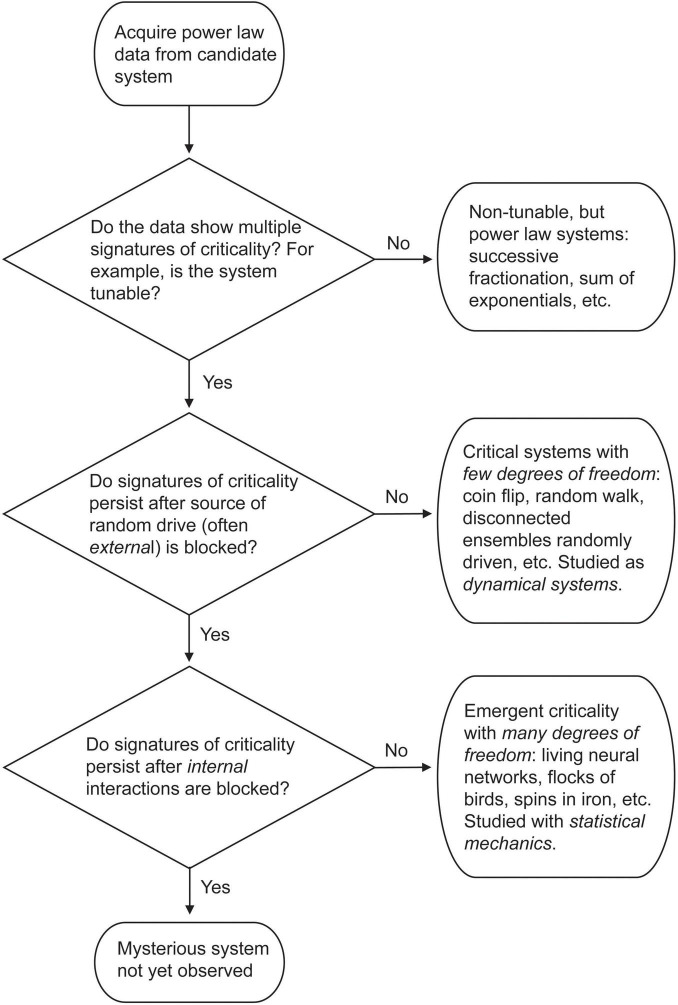
Flow chart describing classification of power law data described in this paper. For explanation, see text.

Figure 15 shows a flow chart for this decision process, starting with the condition that we have some power law data in hand. To properly interrogate the system we must both analyze the data and perform causal interventions, as noted clearly by Priesemann and Shriki in their analysis of this situation (Priesemann and Shriki, 2018).

The first step is to determine if the data show more than just power laws. Do they support the exponent relation and show some type of scaling collapse? Can the process be tuned away from the point where these power laws are produced? Does the system show long-range temporal correlations? If most of these conditions are not satisfied, then the process under consideration is likely to be not critical. We discussed successive fractionation and the sum of many exponential distributions as examples of this category.

The second step is to determine if the signatures of criticality persist after the source of randomness is blocked. Note that this source of randomness can in theory arise from the system’s internal dynamics, but in practice, experimental systems are often driven by external sources of randomness. If blocking this source, whatever its origins, removes signatures of criticality, then the system has few degrees of freedom. These systems are often studied within the framework of dynamical systems. Examples here include the coin flip, a random walk, the Ornstein-Uhlenbeck process when η is small, and possibly swarms of midges.

The third step is to determine if the signatures of criticality persist after internal connections are reduced or cut. If the connections were necessary, then we have a system with emergent criticality. It has many degrees of freedom and is typically studied with the tools of statistical mechanics [but see Dahmen et al. (2019) for a powerful dynamical systems approach to ensembles]. Examples of such systems would include networks of neurons, spins in a piece of iron, or interacting water molecules poised between gas and liquid phases, and possibly murmurations of starlings.

If signatures of criticality persist even after sources of randomness and internal interactions are removed, then we are dealing with a novel type of system that has not to my knowledge been seen.

More broadly, this flow chart is part of a larger process: distinguishing things that are primarily driven by their environment from things that are more autonomous and governed by internal dynamics. This process may eventually be refined to distinguish between things that are living and thinking generators of complexity from things that merely react to external inputs.

We have now come to the end of considering alternatives. Skeptical questions, far from being troublesome, are essential for us to clearly and correctly work through the implications of our experiments. Here, they force us to think carefully about what it means for a network of neurons to operate near criticality and what mechanisms could produce criticality. This in turn helps us to interpret the experiments we need to distinguish between competing models. Those who raise these questions are doing an essential service for science, helping the dialog to go further.

## Author contributions

JB conceived of the study and wrote the manuscript.

## References

[B1] Alstott J. Bullmore E. Plenz D. (2014). powerlaw: a Python package for analysis of heavy-tailed distributions. *PloS one* 9 e85777. 10.1371/journal.pone.0085777 24489671 PMC3906378

[B2] Scarpetta S. Apicella I. I Minati L. de Candia A. (2018). Hysteresis, neural avalanches, and critical behavior near a first-order transition of a spiking neural network. *Physical Review E* 97 062305. 10.1103/PhysRevE.97.062305 30011436

[B3] Arviv O. Goldstein A. Shriki O. (2015). Near-critical dynamics in stimulus-evoked activity of the human brain and its relation to spontaneous resting-state activity. *Journal of Neuroscience* 35 13927–13942. 10.1523/JNEUROSCI.0477-15.2015 26468194 PMC6608180

[B4] Bak P. (1996). *How nature works: the science of self-organized criticality. 1996.* New York: Copurnicus*. 10.1007/978-1-4757-5426-1

[B5] Beggs J. M. (2008). The criticality hypothesis: how local cortical networks might optimize information processing. *Philos Trans A Math Phys Eng Sci* 366 329–343. 10.1098/rsta.2007.2092 17673410

[B6] Beggs J. M. Plenz D. (2003). Neuronal avalanches in neocortical circuits. *J Neurosci* 23 11167–11177. 10.1523/JNEUROSCI.23-35-11167.2003 14657176 PMC6741045

[B7] Beggs J. M. Timme N. (2012). Being critical of criticality in the brain. *Front Physiol* 3 163. 10.3389/fphys.2012.00163 22701101 PMC3369250

[B8] Bellay T. Klaus A. Seshadri S. Plenz D. (2015). Irregular spiking of pyramidal neurons organizes as scale-invariant neuronal avalanches in the awake state. *Elife* 4 e07224. 10.7554/eLife.07224.019 PMC4492006 26151674

[B9] Bertschinger N. Natschlager T. (2004). Real-time computation at the edge of chaos in recurrent neural networks. *Neural Comput* 16 1413–1436. 10.1162/089976604323057443 15165396

[B10] Bienenstock E. (1995). A model of neocortex. *Network: Computation in neural systems* 6 179–224. 10.1088/0954-898X_6_2_004

[B11] Brunel N. (2000). Dynamics of sparsely connected networks of excitatory and inhibitory spiking neurons. *Journal of computational neuroscience* 8 183–208. 10.1023/A:1008925309027 10809012

[B12] Carvalho T. T. Fontenele A. J. Girardi-Schappo M. Feliciano T. Aguiar L. A. Silva T. P. (2020). Subsampled directed-percolation models explain scaling relations experimentally observed in the brain. *arXiv.* preprint*. 10.3389/fncir.2020.576727 33519388 PMC7843423

[B13] Cavagna A. Cimarelli Giardina A. I Parisi G. Santagati R. Stefanini F. Viale M. (2010). Scale-free correlations in starling flocks. *Proceedings of the National Academy of Sciences* 107 11865–11870. 10.1073/pnas.1005766107 20547832 PMC2900681

[B14] Chialvo D. R. (2010). Emergent complex neural dynamics. *Nature physics* 6 744–750. 10.1038/nphys1803

[B15] Chialvo D. R. Bak P. (1999). Learning from mistakes. *Neuroscience* 90 1137–1148. 10.1016/S0306-4522(98)00472-2 10338284

[B16] Chialvo D. R. Cannas S. A. Grigera T. S. Martin D. A. Plenz D. (2020). Controlling a complex system near its critical point *via* temporal correlations. *Scientific reports* 10 1–7. 10.1038/s41598-020-69154-0 32699316 PMC7376152

[B17] Cocchi L. Gollo L. L. Zalesky A. Breakspear M. (2017). Criticality in the brain: A synthesis of neurobiology, models and cognition. *Progress in neurobiology* 158 132–152. 10.1016/j.pneurobio.2017.07.002 28734836

[B18] Cohen M. R. Kohn A. (2011). Measuring and interpreting neuronal correlations. *Nature neuroscience* 14 811–819. 10.1038/nn.2842 21709677 PMC3586814

[B19] Colombo M. A. Wei Y. Ramautar J. R. Linkenkaer-Hansen K. Tagliazucchi E. Van Someren E. J. (2016). More severe insomnia complaints in people with stronger long-range temporal correlations in wake resting-state EEG. *Frontiers in physiology* 7 576. 10.3389/fphys.2016.00576 27965584 PMC5126110

[B20] Dahmen D. Grün S. Diesmann M. Helias M. (2019). Second type of criticality in the brain uncovers rich multiple-neuron dynamics. *Proceedings of the National Academy of Sciences* 116 13051–13060. 10.1073/pnas.1818972116 31189590 PMC6600928

[B21] de Candia A.. Apicella A. I de Arcangelis L. (2021). Critical behaviour of the stochastic Wilson-Cowan model. *PLoS computational biology* 17 e1008884. 10.1371/journal.pcbi.1008884 34460811 PMC8432901

[B22] De Carvalho J. X. Prado C. P. (2000). Self-organized criticality in the Olami-Feder-Christensen model. *Physical review letters* 84 4006. 10.1103/PhysRevLett.84.4006 11019261

[B23] Destexhe A. Touboul J. D. (2021). Is there sufficient evidence for criticality in cortical systems? *Eneuro* 8*. 10.1523/ENEURO.0551-20.2021 33811087 PMC8059881

[B24] Dunkelmann S. Radons G. (1994). “Neural Networsk and Abelian Sandpile Models of Self-Organized Criticality,” in *Proceedings of International Conference Artificial Neural Networks*, (Verlag: Springer).

[B25] Eurich C. W. Herrmann J. M. Ernst U. A. (2002). Finite-size effects of avalanche dynamics. *Physical review E* 66 066137. 10.1103/PhysRevE.66.066137 12513377

[B26] Finlinson K. Shew W. L. Larremore D. B. Restrepo J. G. (2020). Optimal control of excitable systems near criticality. *Physical Review Research* 2 033450. 10.1103/PhysRevResearch.2.033450

[B27] Fontenele A. J. de Vasconcelos N. A. P. Feliciano T. Aguiar L. A. A. Soares-Cunha C. Coimbra B. (2019). Criticality between Cortical States. *Phys Rev Lett* 122 208101. 10.1103/PhysRevLett.122.208101 31172737

[B28] Fosque L. J. Williams-García R. V. Beggs J. M. Ortiz G. (2021). Evidence for quasicritical brain dynamics. *Physical Review Letters* 126 098101. 10.1103/PhysRevLett.126.098101 33750159

[B29] Freeman W. J. (1987). Simulation of chaotic EEG patterns with a dynamic model of the olfactory system. *Biological cybernetics* 56 139–150. 10.1007/BF00317988 3593783

[B30] Friedman N. Ito S. Brinkman B. A. Shimono M. DeVille R. E. Dahmen K. A. (2012). Universal critical dynamics in high resolution neuronal avalanche data. *Phys Rev Lett* 108 208102. 10.1103/PhysRevLett.108.208102 23003192

[B31] Gautam S. H. Hoang T. T. McClanahan K. Grady S. K. Shew W. L. (2015). Maximizing Sensory Dynamic Range by Tuning the Cortical State to Criticality. *PLoS Comput Biol* 11 e1004576. 10.1371/journal.pcbi.1004576 26623645 PMC4666488

[B32] Girardi-Schappo M. Brochini L. Costa A. A. Carvalho T. T. Kinouchi O. (2020). Synaptic balance due to homeostatically self-organized quasicritical dynamics. *Physical Review Research* 2 012042. 10.1103/PhysRevResearch.2.012042

[B33] Greenfield E. Lecar H. (2001). Mutual information in a dilute, asymmetric neural network model. *Physical Review E* 63 041905. 10.1103/PhysRevE.63.041905 11308875

[B34] Haldeman C. Beggs J. M. (2005). Critical branching captures activity in living neural networks and maximizes the number of metastable States. *Phys Rev Lett* 94 058101. 10.1103/PhysRevLett.94.058101 15783702

[B35] Hardstone R. Poil S.-S. Schiavone G. Jansen R. Nikulin V. V. Mansvelder H. D. (2012). Detrended fluctuation analysis: a scale-free view on neuronal oscillations. *Frontiers in physiology* 3 450. 10.3389/fphys.2012.00450 23226132 PMC3510427

[B36] Herz A. V. Hopfield J. J. (1995). Earthquake cycles and neural reverberations: collective oscillations in systems with pulse-coupled threshold elements. *Physical review letters* 75 1222. 10.1103/PhysRevLett.75.1222 10060236

[B37] Hu J. Zheng Y. Gao J. (2013). Long-range temporal correlations, multifractality, and the causal relation between neural inputs and movements. *Frontiers in Neurology* 4 158. 10.3389/fneur.2013.00158 24130549 PMC3793199

[B38] Ihlen E. A. F. E. (2012). Introduction to multifractal detrended fluctuation analysis in Matlab. *Frontiers in physiology* 3 141. 10.3389/fphys.2012.00141 22675302 PMC3366552

[B39] Kauffman S. (1969). Homeostasis and differentiation in random genetic control networks. *Nature* 224 177–178. 10.1038/224177a0 5343519

[B40] Kelso J. (1984). Phase transitions and critical behavior in human bimanual coordination. *American Journal of Physiology-Regulatory, Integrative and Comparative Physiology* 246 R1000–R1004. 10.1152/ajpregu.1984.246.6.R1000 6742155

[B41] Kinouchi O. Copelli M. (2006). Optimal dynamical range of excitable networks at criticality. *Nature physics* 2 348–351.

[B42] Klaus A. Yu S. Plenz D. (2011). Statistical analyses support power law distributions found in neuronal avalanches. *PloS one* 6 e19779. 10.1371/journal.pone.0019779 21720544 PMC3102672

[B43] Kostinski S. Amir A. (2016). An elementary derivation of first and last return times of 1D random walks. *American Journal of Physics* 84 57–60. 10.1119/1.4930092

[B44] Laurson L. Illa X. Santucci S. Tallakstad K. T. Måløy K. J. Alava M. J. (2013). Evolution of the average avalanche shape with the universality class. *Nature communications* 4 1–6. 10.1038/ncomms3927 24352571 PMC3905775

[B45] Lee D. Port N. L. Kruse W. Georgopoulos A. P. (1998). Variability and correlated noise in the discharge of neurons in motor and parietal areas of the primate cortex. *Journal of Neuroscience* 18 1161–1170. 10.1523/JNEUROSCI.18-03-01161.1998 9437036 PMC6792758

[B46] Legenstein R. Maass W. (2007). Edge of chaos and prediction of computational performance for neural circuit models. *Neural networks* 20 323–334. 10.1016/j.neunet.2007.04.017 17517489

[B47] Linkenkaer-Hansen K. Nikouline V. V. Palva J. M. Ilmoniemi R. J. (2001). Long-range temporal correlations and scaling behavior in human brain oscillations. *Journal of Neuroscience* 21 1370–1377. 10.1523/JNEUROSCI.21-04-01370.2001 11160408 PMC6762238

[B48] Lombardi F. Herrmann H. J. Perrone-Capano C. Plenz D. De Arcangelis L. (2012). Balance between excitation and inhibition controls the temporal organization of neuronal avalanches. *Physical review letters* 108 228703. 10.1103/PhysRevLett.108.228703 23003665

[B49] Lombardi F. Herrmann H. J. Plenz D. De Arcangelis L. (2014). On the temporal organization of neuronal avalanches. *Frontiers in systems neuroscience* 8 204. 10.3389/fnsys.2014.00204 25389393 PMC4211381

[B50] Lombardi F. Shriki O. Herrmann H. J. de Arcangelis L. (2021). Long-range temporal correlations in the broadband resting state activity of the human brain revealed by neuronal avalanches. *Neurocomputing* 461 657–666. 10.1016/j.neucom.2020.05.126

[B51] Ma Z. Turrigiano G. G. Wessel R. Hengen K. B. (2019). Cortical Circuit Dynamics Are Homeostatically Tuned to Criticality *In Vivo*. *Neuron 104(4)* 65 e654. 10.1016/j.neuron.2019.08.031 31601510 PMC6934140

[B52] MacLean J. N. Watson B. O. Aaron G. B. Yuste R. (2005). Internal dynamics determine the cortical response to thalamic stimulation. *Neuron* 48 811–823. 10.1016/j.neuron.2005.09.035 16337918

[B53] Mariani B. Nicoletti G. Bisio M. Maschietto M. Oboe R. Leparulo A. (2021). Neuronal avalanches across the rat somatosensory barrel cortex and the effect of single whisker stimulation. *Front. Syst. Neurosci.* 15. 10.3389/fnsys.2021.709677 PMC8435673 34526881

[B54] Mariani B. Nicoletti G. Bisio M. Maschietto M. Vassanelli S. Suweis S. (2022). Disentangling the critical signatures of neural activity. *Sci. Rep.* 12(1), 1–12. 35750684 10.1038/s41598-022-13686-0 PMC9232560

[B55] Marshall N. Timme N. M. Bennett N. Ripp M. Lautzenhiser E. Beggs J. M. (2016). Analysis of Power Laws, Shape Collapses, and Neural Complexity: New Techniques and MATLAB Support *via* the NCC Toolbox. *Front Physiol* 7 250. 10.3389/fphys.2016.00250 27445842 PMC4921690

[B56] Martinello M. Hidalgo J. Maritan A. Di Santo S. Plenz D. Muñoz M. A. (2017). Neutral theory and scale-free neural dynamics. *Physical Review X* 7 041071. 10.1103/PhysRevX.7.041071

[B57] Meisel C. (2020). Antiepileptic drugs induce subcritical dynamics in human cortical networks. *Proceedings of the National Academy of Sciences* 117 11118–11125. 10.1073/pnas.1911461117 32358198 PMC7245074

[B58] Meisel C. Bailey K. Achermann P. Plenz D. (2017a). Decline of long-range temporal correlations in the human brain during sustained wakefulness. *Scientific reports* 7 1–11. 10.1038/s41598-017-12140-w 28928479 PMC5605531

[B59] Meisel C. Klaus A. Vyazovskiy V. V. Plenz D. (2017b). The interplay between long-and short-range temporal correlations shapes cortex dynamics across vigilance states. *Journal of neuroscience* 37 10114–10124. 10.1523/JNEUROSCI.0448-17.2017 28947577 PMC5647769

[B60] Meisel C. Olbrich E. Shriki O. Achermann P. (2013). Fading signatures of critical brain dynamics during sustained wakefulness in humans. *J Neurosci* 33 17363–17372. 10.1523/JNEUROSCI.1516-13.2013 24174669 PMC3858643

[B61] Miller S. R. Yu S. Plenz D. (2019). The scale-invariant, temporal profile of neuronal avalanches in relation to cortical γ–oscillations. *Scientific reports* 9 1–14. 10.1038/s41598-019-52326-y 31712632 PMC6848117

[B62] Mitzenmacher M. (2004). A brief history of generative models for power law and lognormal distributions. *Internet mathematics* 1 226–251. 10.1080/15427951.2004.10129088

[B63] Schwab D. J. Nemenman I Mehta P. (2014). Zipf’s law and criticality in multivariate data without fine-tuning. *Physical review letters* 113 068102. 10.1103/PhysRevLett.113.068102 25148352 PMC5142845

[B64] Nicoletti G. Busiello D. M. (2021). Mutual information disentangles interactions from changing environments. *Physical review letters* 127 228301. 10.1103/PhysRevLett.127.228301 34889638

[B65] Nordlie E. Gewaltig M.-O. Plesser H. E. (2009). Towards reproducible descriptions of neuronal network models. *PLoS computational biology* 5 e1000456. 10.1371/journal.pcbi.1000456 19662159 PMC2713426

[B66] Papanikolaou S. Bohn F. Sommer R. L. Durin G. Zapperi S. Sethna J. P. (2011). Universality beyond power laws and the average avalanche shape. *Nature Physics* 7 316–320. 10.1038/nphys1884

[B67] Petermann T. Thiagarajan T. C. Lebedev M. A. Nicolelis M. A. Chialvo D. R. Plenz D. (2009). Spontaneous cortical activity in awake monkeys composed of neuronal avalanches. *Proceedings of the National Academy of Sciences* 106 15921–15926. 10.1073/pnas.0904089106 19717463 PMC2732708

[B68] Poil S.-S. Hardstone R. Mansvelder H. D. Linkenkaer-Hansen K. (2012). Critical-state dynamics of avalanches and oscillations jointly emerge from balanced excitation/inhibition in neuronal networks. *Journal of Neuroscience* 32 9817–9823. 10.1523/JNEUROSCI.5990-11.2012 22815496 PMC3553543

[B69] Poil S. S. van Ooyen A. Linkenkaer-Hansen K. (2008). Avalanche dynamics of human brain oscillations: relation to critical branching processes and temporal correlations. *Human brain mapping* 29 770–777. 10.1002/hbm.20590 18454457 PMC6871218

[B70] Ponce-Alvarez A. Jouary A. Privat M. Deco G. Sumbre G. (2018). Whole-Brain Neuronal Activity Displays Crackling Noise Dynamics. *Neuron 100(6)* 144 e1446. 10.1016/j.neuron.2018.10.045 30449656 PMC6307982

[B71] Priesemann V. Shriki O. (2018). Can a time varying external drive give rise to apparent criticality in neural systems? *PLoS computational biology* 14 e1006081. 10.1371/journal.pcbi.1006081 29813052 PMC6002119

[B72] Priesemann V. Valderrama M. Wibral M. Van Quyen M. Le (2013). Neuronal avalanches differ from wakefulness to deep sleep–evidence from intracranial depth recordings in humans. *PLoS computational biology* 9*. 10.1371/journal.pcbi.1002985 23555220 PMC3605058

[B73] Reed W. J. Hughes B. D. (2002). From gene families and genera to incomes and internet file sizes: Why power laws are so common in nature. *Physical Review E* 66 067103. 10.1103/PhysRevE.66.067103 12513446

[B74] Sethna J. P. Dahmen K. A. Myers C. R. (2001). Crackling noise. *Nature* 410 242–250. 10.1038/35065675 11258379

[B75] Shew W. L. Clawson W. P. Pobst J. Karimipanah Y. Wright N. C. Wessel R. (2015). Adaptation to sensory input tunes visual cortex to criticality. *Nature Physics* 11*. 10.1038/nphys3370

[B76] Shew W. L. Plenz D. (2013). The functional benefits of criticality in the cortex. *Neuroscientist* 19 88–100. 10.1177/1073858412445487 22627091

[B77] Shew W. L. Yang H. Petermann T. Roy R. Plenz D. (2009). Neuronal avalanches imply maximum dynamic range in cortical networks at criticality. *J Neurosci* 29 15595–15600. 10.1523/JNEUROSCI.3864-09.2009 20007483 PMC3862241

[B78] Shew W. L. Yang H. Yu S. Roy R. Plenz D. (2011). Information capacity and transmission are maximized in balanced cortical networks with neuronal avalanches. *J Neurosci* 31 55–63. 10.1523/JNEUROSCI.4637-10.2011 21209189 PMC3082868

[B79] Shriki O. Alstott J. Carver F. Holroyd T. Henson R. N. Smith M. L. (2013). Neuronal avalanches in the resting MEG of the human brain. *Journal of Neuroscience* 33 7079–7090. 10.1523/JNEUROSCI.4286-12.2013 23595765 PMC3665287

[B80] Spasojević D. Bukvić S. Milošević S. Stanley H. E. (1996). Barkhausen noise: Elementary signals, power laws, and scaling relations. *Physical Review E* 54 2531. 10.1103/PhysRevE.54.2531 9965364

[B81] Spitzner F. Dehning J. Wilting J. Hagemann A. Neto J. Zierenberg J. (2020). MR. Estimator, a toolbox to determine intrinsic timescales from subsampled spiking activity. *arXiv.* preprint*. 10.1371/journal.pone.0249447 33914774 PMC8084202

[B82] Tapiero C. S. Vallois P. (1996). Run length statistics and the Hurst exponent in random and birth-death random walks. *Chaos, Solitons & Fractals* 7 1333–1341. 10.1016/0960-0779(96)00032-X

[B83] Timme N. M. Marshall N. J. Bennett N. Ripp M. Lautzenhiser E. Beggs J. M. (2016). Criticality Maximizes Complexity in Neural Tissue. *Front Physiol* 7 425. 10.3389/fphys.2016.00425 27729870 PMC5037237

[B84] Touboul J. Destexhe A. (2017). Power-law statistics and universal scaling in the absence of criticality. *Physical Review E* 95 012413. 10.1103/PhysRevE.95.012413 28208383

[B85] van der Vaart K. Sinhuber M. Reynolds A. M. Ouellette N. T. (2020). Environmental perturbations induce correlations in midge swarms. *Journal of the Royal Society Interface* 17 20200018. 10.1098/rsif.2020.0018 32208820 PMC7115240

[B86] Villegas P. di Santo S. Burioni R. Muñoz M. A. (2019). Time-series thresholding and the definition of avalanche size. *Physical Review E* 100 012133. 10.1103/PhysRevE.100.012133 31499802

[B87] Williams-Garcia R. V. Moore M. Beggs J. M. Ortiz G. (2014). Quasicritical brain dynamics on a nonequilibrium Widom line. *Phys Rev E Stat Nonlin Soft Matter Phys* 90 062714. 10.1103/PhysRevE.90.062714 25615136

[B88] Wilson H. R. Cowan J. D. (1972). Excitatory and inhibitory interactions in localized populations of model neurons. *Biophysical journal* 12 1–24. 10.1016/S0006-3495(72)86068-5 4332108 PMC1484078

[B89] Wilting J. Priesemann V. (2018). Inferring collective dynamical states from widely unobserved systems. *Nature communications* 9 1–7. 10.1038/s41467-018-04725-4 29899335 PMC5998151

[B90] Wilting J. Priesemann V. (2019). Between perfectly critical and fully irregular: A reverberating model captures and predicts cortical spike propagation. *Cerebral Cortex* 29 2759–2770. 10.1093/cercor/bhz049 31008508 PMC6519697

[B91] Worrell G. A. Cranstoun S. D. Echauz J. Litt B. (2002). Evidence for self-organized criticality in human epileptic hippocampus. *Neuroreport* 13 2017–2021. 10.1097/00001756-200211150-00005 12438917

