# Supplementary material for: Addressing skepticism of the critical brain hypothesis
Source: Front Comput Neurosci. 2022 Sep 15;16:703865. doi: 10.3389/fncom.2022.703865 (PMC9520604; doi:10.3389/fncom.2022.703865)
Supplement: Supplementary file 1 [file Data_Sheet_1.zip › SupplementaryMaterial4_Updated/DescriptionOfMethodsAndCodeForFiguresFinalThumbDrive.docx]

**Supplementary material**

This document first contains a description of the main methods used in the paper for the computational demonstrations. This is followed by a description of the Matlab programs used to produce some of the figures. These programs can be found in the zipped folder provided.

**Methods**

**The Brunel model**:

This model has been widely cited in the literature as a canonical description of the dynamics produced by sparsely-connected, integrate and fire neurons as would be found in the mammalian cortex. A detailed analytic description of the model can be found in the seminal paper by Brunel (Brunel, 2000). The computational version of the model used in this paper was adapted from the Matlab code that was posted by (Destexhe and Touboul 2021), which can be found here: <https://zenodo.org/record/4591877> In what follows, I will give a verbal overview of the model with some descriptions of code.

*Architecture and connectivity*: The architecture of the model is given in Figure 3 A. It consists of populations of spiking excitatory neurons (E) and inhibitory neurons (I), driven by an excitatory, spiking external source (E_ext_). Neurons within the E and I populations are randomly connected to each other and to themselves at 10% sparsity; they receive input from E_ext_ without giving feedback to it. Eighty percent of the neurons are excitatory, and twenty percent of the neurons are inhibitory, approximating what has been reported for proportions of E and I neurons in the cortex. In the implementation used for this paper, the connections are all given a set delay of 1.8 ms of simulated time, a setting that Brunel featured. In this implementation, time is discrete and not continuous. Each step represents 0.1 ms of real time and is the time scale at which variables are updated. Most simulations were conducted with the standard 1000 neuron model. In some simulations, this size was changed (e.g., 250 or 8000). Excitatory connections are positive, with initial strength set at 1.2, but divided by the number of neurons to prevent excessive strength as model sizes are changed. Inhibitory connections are all set to be negative one.

*Integrate and fire dynamics*: The neurons are spiking and have leaky integrate and fire dynamics. Each neuron has a voltage threshold of 20 mV, a reset potential of 10 mV, a time constant of 20 ms, and a refractory period of 2 ms. Briefly, the voltage of a neuron was the sum of all its inputs multiplied by their synaptic weights, plus the external drive. When this exceeded threshold, the neuron would fire a spike. After that, the voltage was reset, and it would not fire for the duration of the refractory period. Subthreshold voltage changes would exponentially decay with time constant tau, and could accumulate to drive the neuron over threshold, giving neurons a leaky integrate and fire property. This line of code implements the brief verbal description just given:

V=(1-dt/tau)*V + ExInput(:,1+mod(i-1,1e4)) + W*Rasterplot(:,max(1,i-N_del));

Through this line of code, the external drive has access to every neuron in the network. Types of external drive are described more below.

**Sample Brunel model code**:

Below is the code provided by Touboul and Destexhe in their 2021 paper for simulating the Brunel model. I have highlighted in yellow the portion of the code that determines the external drive. The external drive was changed for some computational demonstrations in this paper.

% Simulation of the Brunel JCNS 2000 network with electrode recording

% spikes (Destexhe, Touboul - PRL Comment, 2020).

% (C) Touboul J.

% jonathan.touboul@gmail.com.

Spikes={};

if ~exist('N_iter')

N_iter=1;

end

for Iteration=1:N_iter

fprintf(sprintf('Iteration %d/%d\n',Iteration,N_iter))

Duration=5; % Total duration

dt=0.1e-3; % time-step

N=1000; % Number of neurons

NTimes=ceil(Duration/dt); % Number of time-steps

%%%%%%%% PARAMETERS OF THE NETWORK %%%%%%%%

NE=ceil(0.8*N); % # excitatory neurons

NI=N-NE; % # excitatory neurons

% (p.185, 2nd column, Brunel 2000)

V_t=20e-3; % voltage threshold

V_r=10e-3; % voltage reset

tau=20e-3; % voltage time constant

t_rp=2e-3; % refractory period

%%%%%% BIFURCATION PARAMETERS in Brunel 2000 %%%%%%%

J=0.12e-3; % Strength of exc. Connections

J=J*10000/N; % Rescaling to make behaviors independent of N

Connectivity=0.1; % Connectivity coefficients, parameter epsilon in Brunel 2000.

D=1.8e-3; % Transmission Delay

CE=round(Connectivity*NE); % Number of exc connections

CI=round(Connectivity*NI); % Number of inh connections

C_ext=CE;

nu_thresh=V_t/(J*CE*tau); % Frequency needed for a neuron to reach the threshold.

%%%%%%%% SIMULATION PARAMETERS %%%%%%%%

N_rp=round(t_rp/dt); % Refractory (in dt)

N_del=round(D/dt); % delay (in dt)

g=4+4*(rand()); % Strength of inh/exc (inh connections -gJ)

ratioextthresh=1.1+0.2*(rand()-0.5); % nu_ext/nu_thresh

nu_ext=ratioextthresh*nu_thresh; % external Poisson input rate

W=zeros(N,N);

%%%%%%%% Generation of the connectivity matrix %%%%%%%%

for i=1:N

ECells=randperm(NE);

ECells=ECells(1:CE);

W(i,ECells)=J;

ICells=randperm(NI);

ICells=NE+ICells(1:CI);

W(i,ICells)=-1;

end

W(W<0)=-g*J;

Rasterplot=zeros(N,NTimes);

Rasterplot_pert=zeros(N,NTimes);

V=V_r*ones(N,1); % Voltage vector

LS=-(N_del+1)*rand(N,1);

allspikes=zeros(1,NTimes);

i=0;

while i<NTimes

i=i+1;

if (1+mod(i-1,1e4))==1

ExInput=J*poissrnd(nu_ext*C_ext*dt,N,1e4);

end

V=(1-dt/tau)*V+ExInput(:,1+mod(i-1,1e4))+W*Rasterplot(:,max(1,i-N_del)); % Voltage update

spike=V>=V_t; % Spiking neurons have a "1"

V(LS>i-N_del)=V_r; % Refractory period.

LS(spike)=i; % Time of last spike

V(spike)=V_r; % Reset membrane potential

% Store spike times

Rasterplot(:,i)=spike; % Each row is (neuron number,spike time)

allspikes(1,i)=sum(spike);

% progressbar(i,NTimes);

end

Spikes{Iteration}=allspikes;

end

A sample plot of some output from this code is given here:


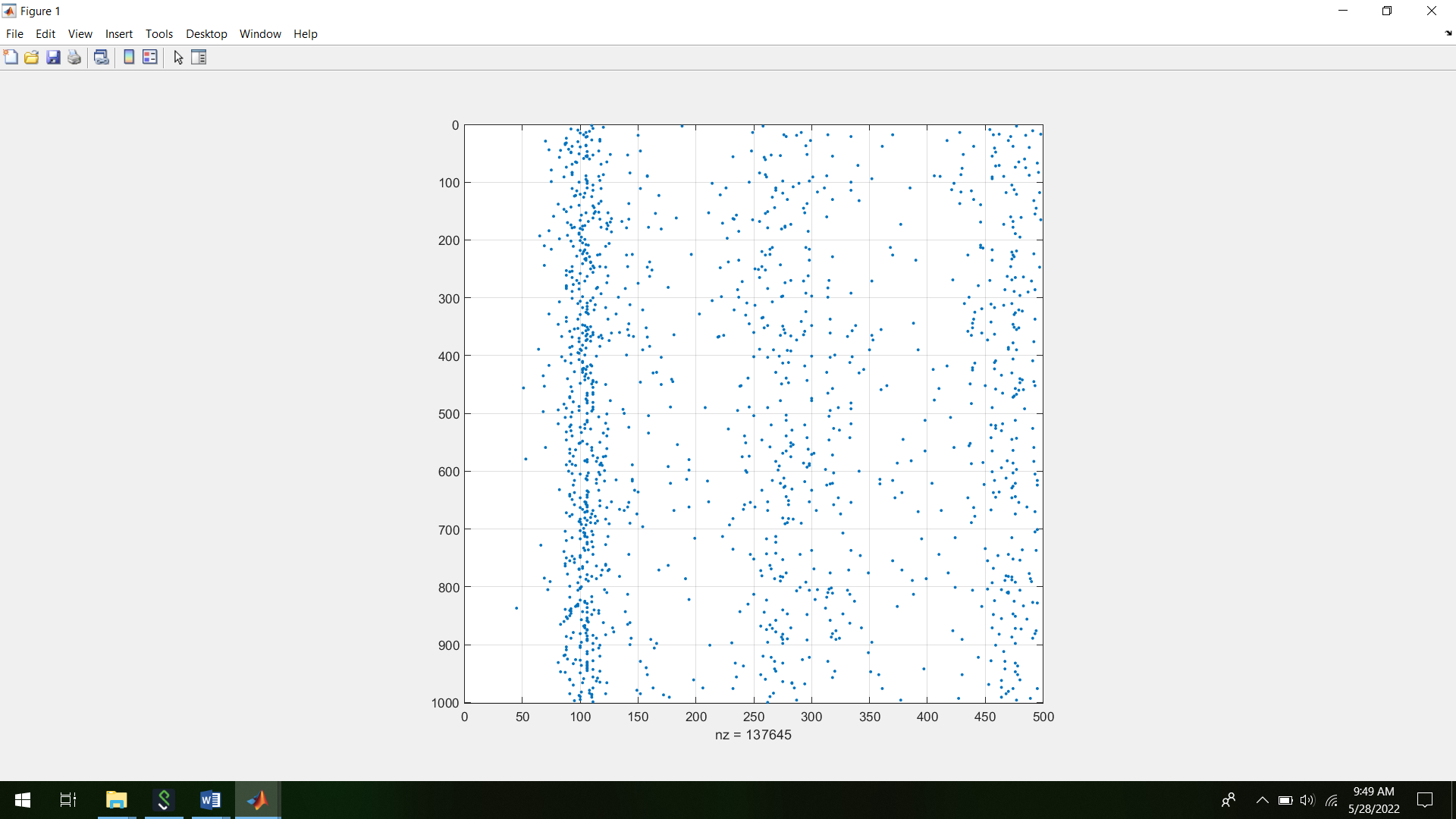


**Tuning the model** **through changes in inhibition:**

As described in the text of the paper, the model could be tuned in various ways. The two ways explored in this paper involved (1) changing the relative strength of inhibition, and (2) changing the relative strength of external drive. These ways are described schematically in Figure 3 B by the red and blue arrows, respectively.

In the original code provided by (Touboul and Destexhe, 2021), the value of g was set to be near 4 with some random variation, as seen in this line:

g=4+4*(rand()); % Strength of inh/exc (inh connections -gJ)

In most of the simulations in this paper, g was controlled and not randomly selected. For example, g would be marched from 0 to 5 in steps of 0.1.

**Tuning the model through changes of external drive**:

I will now describe the different types of external drive that were considered. As mentioned above, increasing external drive is equivalent to moving along the blue arrow shown in Figure 3 B.

(A) In Touboul and Destexhe’s original implementation, this drive was drawn from a Poisson distribution with a given mean value. Each time step, a different value of drive could be drawn, but all would be from a distribution with the same mean; there were no negative values. Every 10000 time steps, however, the mean value of the Poissonian distribution was changed. This resulted in all neurons being driven by the same external drive at all time steps. This drive would randomly change in strength, moving like a random walk but always staying above the time axis and never going below it. Because all neurons were driven by this same strength, it was a form of correlated noise.

(B) To simulate uncorrelated noise, I created an external drive that affected each neuron independently with a probability Ps (for P spontaneous). Ps varied over these values: 0.5, 0.1, 0.01, 0.001. This was the probability of each neuron becoming spontaneously active at each time step.

(C) To simulate correlated noise, I used a rectified coin flip process, explained more below, to drive different numbers of neurons. This rectified coin flip process was conceptually similar to the Poissonian noise described above and was in fact used by Touboul and Destexhe as well, where they demonstrated it produced similar effects to the Poissonian drive (Touboul and Destexhe, 2017). For a 1000 neuron network, the number of neurons driven varied over these values: 10, 100, 500, 1000. The rectified coin flip process is described verbally in the text; here I provide a section of the code with comments:

%create the random walk from a simulated coin flip

steps = randn(N, 1);

steps = sign(steps);

X = cumsum(steps); %Position in a rand walk is cumulative sum of the steps

clear steps

X = abs(X); %a reflected random walk; easier to deal with excursion sizes

First, a random string of numbers is produced. Second, the signs of those numbers are used to determine positive or negative steps of size +1 or -1, respectively. Third, the cumulative sum of these steps is produced, which gives the position of the random walker. Fourth, all negative excursions are rectified so only positive drive is given to the Brunel model.

(D) To measure mutual information through the network, I stimulated it at one chosen time (for example, after 20 time steps) and then measured the network response. In a 1000 neuron network, stimulation took on eight possible values in terms of number of neurons driven to spike at the prescribed time: 0, 125, 250, 375, 500, 625, 750, 875.

**Measuring the time constant**:

In Figures 5, 6 and 13 of the paper, the time constant of the Brunel model was measured in response to stimulation at a given time. This was done under different values of g or different forms of external drive.

To perform these measurements, a fraction of the total number of neurons was stimulated at one given time and then the activity in the network was allowed to evolve over time. For a network of 1000 neurons, for example, 200 neurons would be turned on to fire spikes at time step 20. As shown in Figure 5, such stimulation typically produced three types of responses, depending on the relative strength of inhibition. First, activity could rapidly amplify; second, it could very gradually decay; third, it could rapidly decay. To measure these decay rates, the total activity in the network was sampled at integer numbers times the synaptic delay (1.8 ms). These integer numbers typically ranged from 1 to 30. For a given value of g, the relative inhibitory strength, the model was run 20 times and the average population activity was plotted with standard deviations. From these population activity data over 30 intervals of the synaptic delay, an exponential curve was fit using the Matlab curve fitting toolbox. The time constant of each curve was reported

**Measuring mutual information and response entropy**:

Mutual information was measured with this standard equation: MI(S;R) = H(R) – H(R|S), where S is the stimulus, R is the response, H(R) is the entropy of the response and H(S|R) is the entropy of the response conditioned on the stimulus. The code for accomplishing this is given in the Matlab function Brunel_Renormalization.m. Here I will give an overview of how that program in works.

The stimulus, S was merely the number of neurons that were stimulated. As mentioned before, this could be (0, 125, 250, 375, 500, 625, 750, 875), spanning 8 values, or 3 bits (8 = 2^3^). These values were always the same and needed no averaging.

The response, R, was merely the average number of neurons active over a range of synaptic delay steps, for example 3 through 5, after the stimulation event. To create a probability distribution from these responses, the following steps were used. First, the maximum and minimum response values were found. Second, this range was partitioned into equal intervals. The number of times a response was produced in each interval was noted. Third, the number of responses in each interval was divided by the total number of responses, yielding a probability of response for each interval, p_i_.

Entropy of the response, H(R), was measured in the standard way:

$$H(R) = -\sum_{i=1}^{n} p_{i}{\times log}_{2}(p_{i})$$

Here p_i_ is the probability of the ith response and n is the total number of intervals in the response distribution.

The entropy of the response conditioned on the stimulus, H(R|S) was produced in several steps. First, a matrix was created with the number of rows equal to the number of types of stimuli delivered (here 8), and the number of columns equal to the number of types of responses produced. Second, this matrix was filled by counting how often each response was produced by each stimulus. This was normalized so that each row represented the probability of each response to that given stimulus. Third, the entropy of each row was calculated using the formula for entropy given above, and these entropies were then summed up.

To calculate mutual information, the entropy of the response conditioned on the stimulus was merely subtracted from the entropy of the response.

**Power law fitting**:

All candidate power laws were subjected to statistical tests. Our lab group produced a Matlab toolbox for this and other functions related to assessing criticality in neural data. The code is included in the supplementary materials as a zipped folder. These methods are described in detail in the following paper:

Marshall, N., N. M. Timme, N. Bennett, M. Ripp, E. Lautzenhiser and J. M. Beggs (2016). "Analysis of Power Laws, Shape Collapses, and Neural Complexity: New Techniques and MATLAB Support via the NCC Toolbox." Front Physiol **7**: 250.

**Code**

**Matlab codes corresponding to Figures in the paper**:

**Figure 2**: Both plots are produced by the script RandomWalker.m. The script calls polyfit.m and polyval.m from the Matlab Curve Fitting Toolbox.

**Figure 5**: The script BrunelTimeConstantFinal.m calls the function BrunelNetworkFunctionTimeConstantFinal.m which calls the function fit.m from the Matlab Curve Fitting Toolbox. Running BrunelTimeConstantFinal.m will produce a set of plots very similar to those shown in the figure. This will give the time constants for a Brunel network model with 1000 neurons.

**Figure 6**: BrunelTimeConstantLarge.m calls BrunelNetworkFunctionTimeConstantLarge.m to produce a plot of time constants over a limited range of g values. To span different g values, just change the entries in the g vector in line 16 of the code. This will give the time constants for a Brunel network model with 8000 neurons. Because of the increased size, it will take longer to run.

**Figure 7**: BrunelScript5_2000.m is a batch job that plots mutual information for varying values of g for a network size of 2000 neurons and saves each file. It calls BrunelNetworkFunction5.m, DynamicRangeFunction.m and Brunel_Renormalization.m. You can change the network sizes to 1500, 1000, 500 or 250 by changing the variable NumNeurons in the script, which first appears on line 6, then again on line 107, and so on for each run of the model. After multiple sizes of network are run, you can fit an exponential curve to the data and extract the asymptotic value as well as 95% confidence bounds using the function fit.m from the Matlab Curve Fitting Toolbox.

**Figure 9**: The Ornstein-Uhlenbeck simulation is run with OrnsteinUhlenbeckScriptDemo.m It plots the variable over time, as well as distributions for avalanche sizes, durations and avg. size vs. duration.

**Figure 10**: BrunelScript7.m is a batch job to plot the mutual information curves for networks with varying levels of spontaneous, uncorrelated noise; it saves each file. It calls BrunelNetworkFunction6.m, DynamicRangeFunction.m and Brunel_Renormalization.m.

**Figure 11**: BrunelScript8.m is a batch job to plot the mutual information curves with varying levels of spontaneous, correlated noise; it saves each file. It calls BrunelNetworkFunction6.m, BrunelNetworkFunction8.m, CoinFlipNoise.m, DynamicRangeFunction.m and Brunel_Renormalization.m.

**Figure 12**: The coin flip simulation is generated by the script CoinFlipFinal.m. If you run it for many steps (2 billion or more produces the best plots), you need sufficient RAM. Insufficient RAM will lead to error statements “Matlab is out of memory.” You can then set the number of steps to be fewer, but the results will not be as clear. If you cannot run it for 2 billion time steps, I have found that just running it with 200 million steps several times (say 5 – 10) usually produces one good run where the process returns to the axis enough times to produce good plots. Since it is probabilistic, there are occasions where the random walk diverges and does not return for extremely long times. On these occasions, the statistics will be very poor.

To test statistical significance, use PowerLawSignificance.m, which calls on functions from the freeware Matlab toolbox NCCToolboxV1 from the following paper:

Marshall, N., N. M. Timme, N. Bennett, M. Ripp, E. Lautzenhiser and J. M. Beggs (2016). "Analysis of Power Laws, Shape Collapses, and Neural Complexity: New Techniques and MATLAB Support via the NCC Toolbox." Front Physiol **7**: 250.

PowerLawSignificance.m will output a p value; if it is greater than 0.20, then the distribution is more likely to be fit by a power law function than other candidate functions.

**Figure 13 A**: Two avalanche size distributions produced by the Brunel model, under some spontaneous drive to generate avalanches, for two different levels of connectivity (10% and 5%): BrunelDemoFinal.m is the script. It calls BrunelNetworkFunction.m, runstatsAvalanches.m. This requires that you have the Statistics and Machine Learning toolbox. The program BrunelNetworkFunction.m can require a lot of memory; the default setting for the variable Duration is 50 (see line 26), but if you get a memory error message, try making it smaller, e.g., Duration = 20. That should help.

**Figure 13 B**: To see how the peak in the time constant plot is diminished by reduced connectivity, run BrunelTimeConstantConnectivityChange.m, which is just like BrunelTimeConstant.m but varies the connectivity density from 10% down to 1%. Calls BrunelNetworkFunctionTimeConstant2.m

**Figure 13 C**: Two mutual information plots are produced for Brunel models with 10% and 1% connectivity. The script is MIconnectivityScript.m. It calls BrunelNetworkSimulationFunction4.m and Brunel_Renormalization.m.

All of these codes are contained in the zipped folder uploaded in the supplementary materials. In addition, I have included a folder DestexheTouboulCode that contains the programs freely offered by Jonathan Touboul and Alain Destexhe. The folder also contains the NCC Toolbox for fitting avalanche data and measuring the probability that they were drawn from power law distributions. Access to folders should be provided by giving an addpath command in the Matlab Command Window. For example: addpath C:\Users\jmbeggs\MatlabWork\NickSoftware\NCCToolboxV1. Your folder structure may be different, of course.
